# Supplementary material for: Mono- and Dimeric Sorbicillinoid Inhibitors Targeting IL-6 and IL-1β from the Mangrove-Derived Fungus Trichoderma reesei BGRg-3
Source: Int J Mol Sci. 2023 Nov 8;24(22):16096. doi: 10.3390/ijms242216096 (PMC10670970; doi:10.3390/ijms242216096)
Supplement: Supplementary file 1 [file ijms-24-16096-s001.zip › ijms-2705268-supplementary.pdf]

Mono- and Dimeric Sorbicillinoid Inhibitors Targeting the IL-6 and IL-1β from the Mangrove-Derived Fungus *Trichoderma reesei* BGRg-3

Yufeng Liu[a,1], Tao Chen[a,1], Bing Sun[a], Qi Tan[a], Hui Ouyang[b], Bo Wang[a,], Huijuan Yu[b,*], Zhigang She[a,*]

[a] School of Chemistry, Sun Yat-Sen University, Guangzhou 510275, China; liuyf76@mail2.sysu.edu.cn (Y.L.); chent296@mail2.sysu.edu.cn (T.C.); sunb33@mail.sysu.edu.cn (B.S.); tanq27@mail2.sysu.edu.cn (Q.T.); ouyh0807@gmail.com (H.O.); ceswb@mail.sysu.edu.cn (B.W.)

[b] Guangdong Key Laboratory of Animal Conservation and Resource Utilization, Guangdong Public Laboratory of Wild Animal Conservation and Utilization, Institute of Zoology, Guangdong Academy of Sciences, Guangzhou 510260, China

* Correspondence: yuhj@giz.gd.cn (H.Y.); cesshzhg@mail.sysu.edu.cn (Z.S.)

[1] Authors contributed equally to this work.

Supporting information

Fig.S1. HRESIMS spectrum of compound **1** 4

Fig.S2. ^{1}H NMR spectrum of compound **1** (600 MHz, CD_3OD) 4

Fig.S3. ^{13}C NMR spectrum of compound **1** (600 MHz, CD_3OD) 5

Fig.S4. DEPT135 spectrum of compound **1** (400 MHz, CD_3OD) 5

Fig.S5. HSQC spectrum of compound **1** (CD_3OD) 6

Fig.S6. H,H-COSY spectrum of compound **1** (CD_3OD) 6

Fig.S7. HMBC spectrum of compound **1** (CD_3OD) 7

Fig.S8. NOE spectrum of compound **1** (CD_3OD) 7

Fig.S9. HRESIMS spectrum of compound **2** 8

Fig.S10. ^{1}H NMR spectrum of compound **2** (400 MHz, CD_3OD) 8

Fig.S11. ^{13}C NMR spectrum of compound **2** (400 MHz, CD_3OD) 9

Fig.S12. DEPT135 spectrum of compound **2** (400 MHz, CD_3OD) 10

Fig.S13. HSQC spectrum of compound **2** (CD_3OD) 10

Fig.S14. H,H-COSY spectrum of compound **2** (CD_3OD) 10

Fig.S15. HMBC spectrum of compound **2** (CD_3OD) 11

Fig.S16. NOE spectrum of compound **2** (CD_3OD) 11

Fig.S17. HRESIMS spectrum of compound **3** 12

Fig.S18. ^{1}H NMR spectrum of compound **3** (400 MHz, $CDCl_3$) 12

Fig.S19. ^{13}C NMR spectrum of compound **3** (400 MHz, $CDCl_3$) 13

Fig.S20. DEPT135 spectrum of compound **3** (400 MHz, $CDCl_3$) 13

Fig.S21. HSQC spectrum of compound **3** ($CDCl_3$) 14

Fig.S22. H,H-COSY spectrum of compound **3** ($CDCl_3$) 14

Fig.S23. HMBC spectrum of compound **3** ($CDCl_3$) 15

Fig.S24. NOE spectrum of compound **3** ($CDCl_3$) 15

Fig.S25. HRESIMS spectrum of compound **5** 16

Fig.S26. ^{1}H NMR spectrum of compound **5** (400 MHz, $CDCl_3$) 16

Fig.S27. ^{13}C NMR spectrum of compound **5** (400 MHz, $CDCl_3$) 17

Fig.S28. DEPT135 spectrum of compound **5** (400 MHz, $CDCl_3$) 17

Fig.S29. HSQC spectrum of compound **5** ($CDCl_3$) 18

Fig.S30. H,H-COSY spectrum of compound **5** ($CDCl_3$).......... 18

Fig.S31. HMBC spectrum of compound **5** ($CDCl_3$) 19

Fig.S32. NOE spectrum of compound **5** ($CDCl_3$) 19

Fig.S33. UV and ECD of compound **1** (CH_3OH) 20

Fig.S34. UV of compound **2** (CH_3OH) 20

Fig.S35. UV and ECD of compound **3** (CH_3OH) 20

Fig.S36. UV and ECD of compound **5** (CH_3OH) 21

Fig.S37. Isolation procedure 21

Biosynthesis pathway 21

Fig.S38. Biosynthesis pathway of isolated mono-sorbicillinoids 22

Fig.S39. Biosynthesis pathway of isolated dimeric sorbicillinoids.......... 23

ECD calculations data of **1** 23

ECD calculations data of **3** 29

ECD calculations data of **5** 43

Reference.......... 47

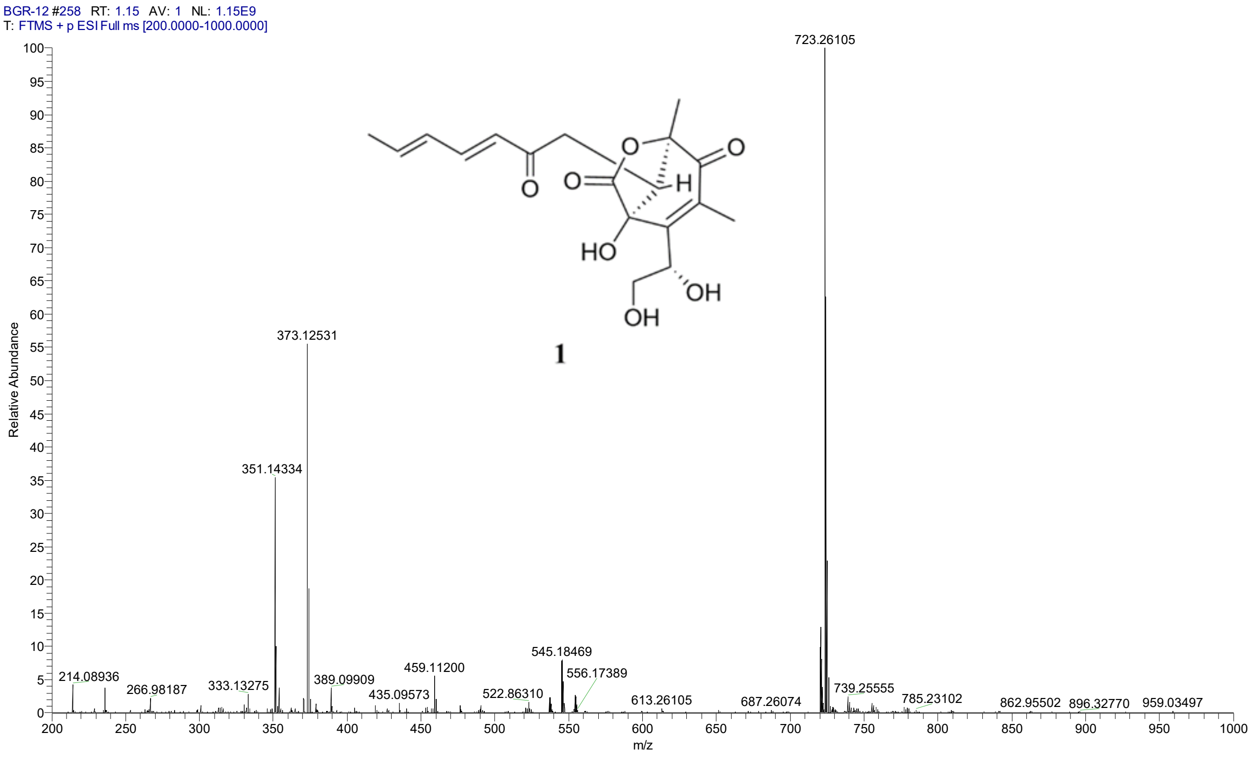

Fig.S1. HRESIMS spectrum of compound **1**

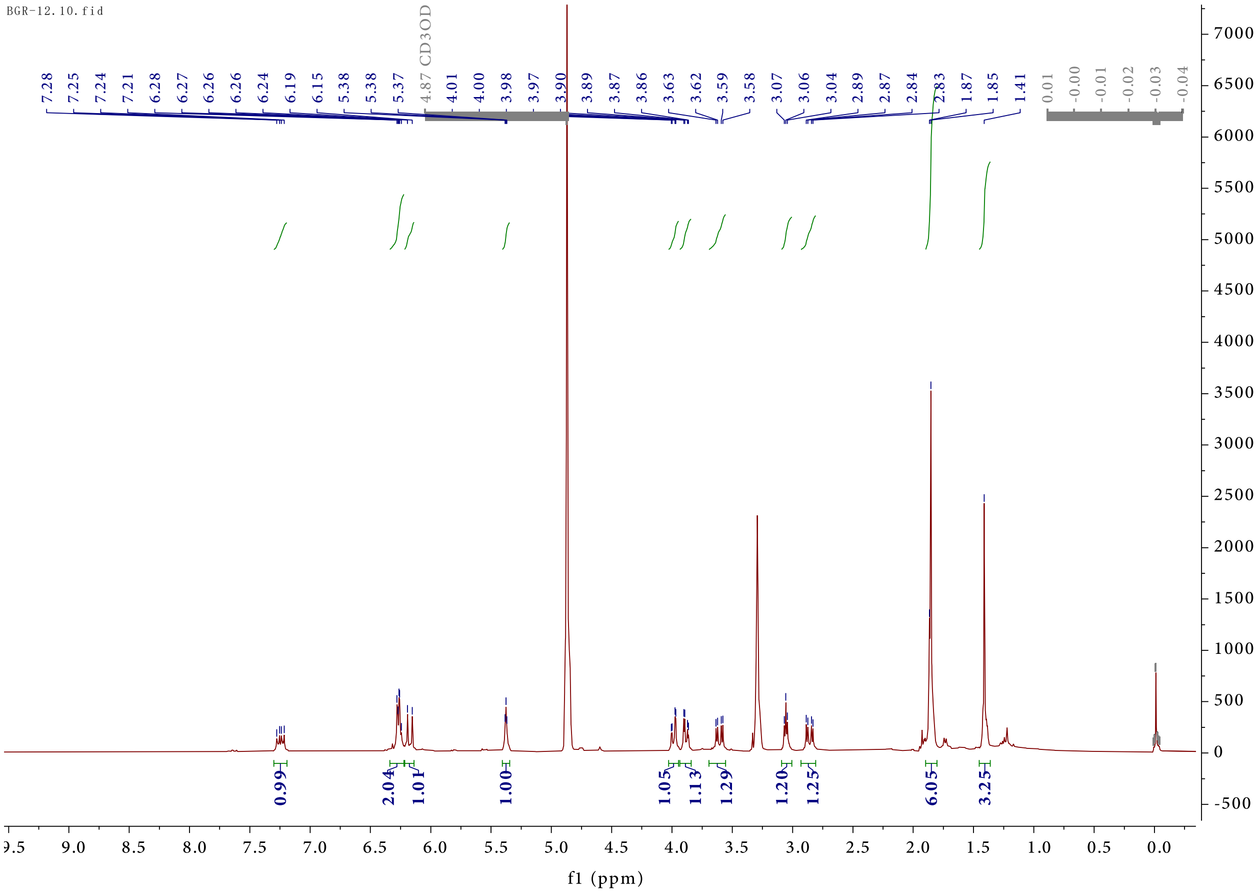

Fig.S2. ^{1}H NMR spectrum of compound **1** (600 MHz, CD_3OD)

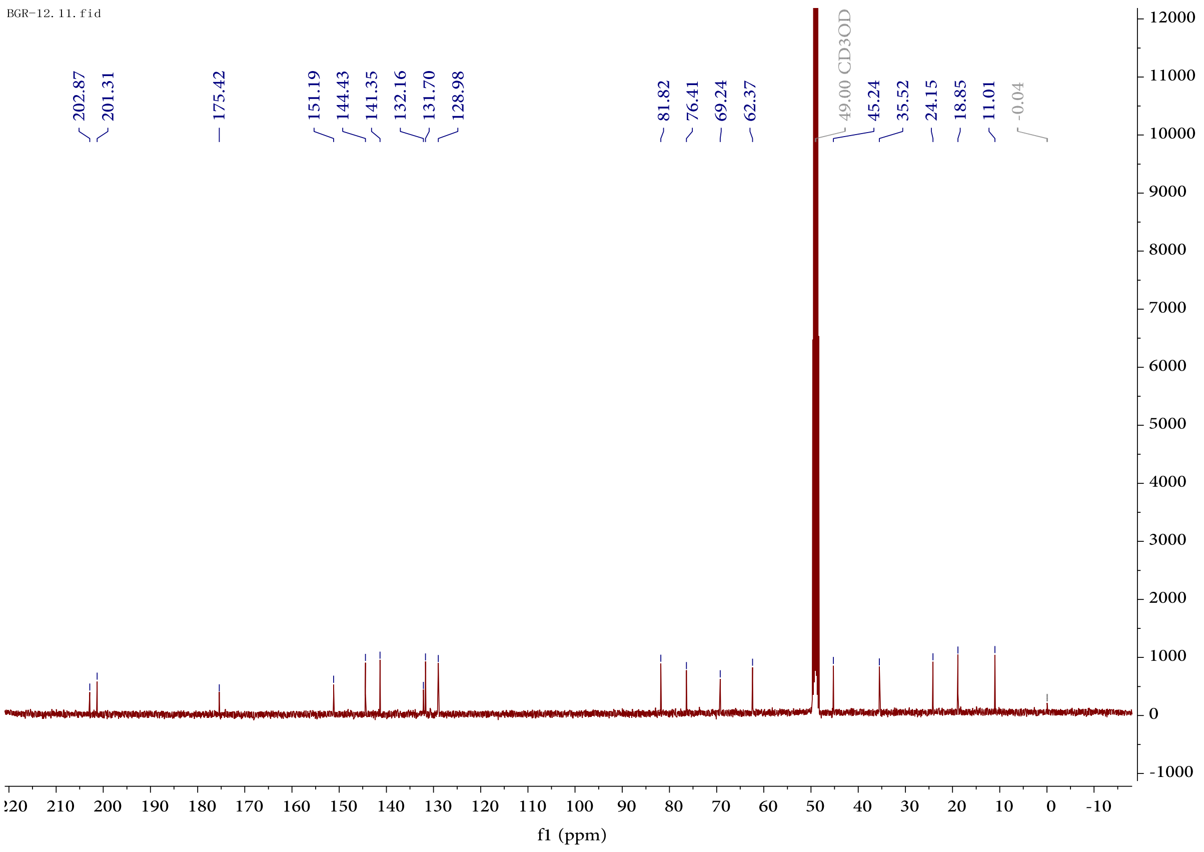

Fig.S3. ^{13}C NMR spectrum of compound **1** (600 MHz, CD_3OD)

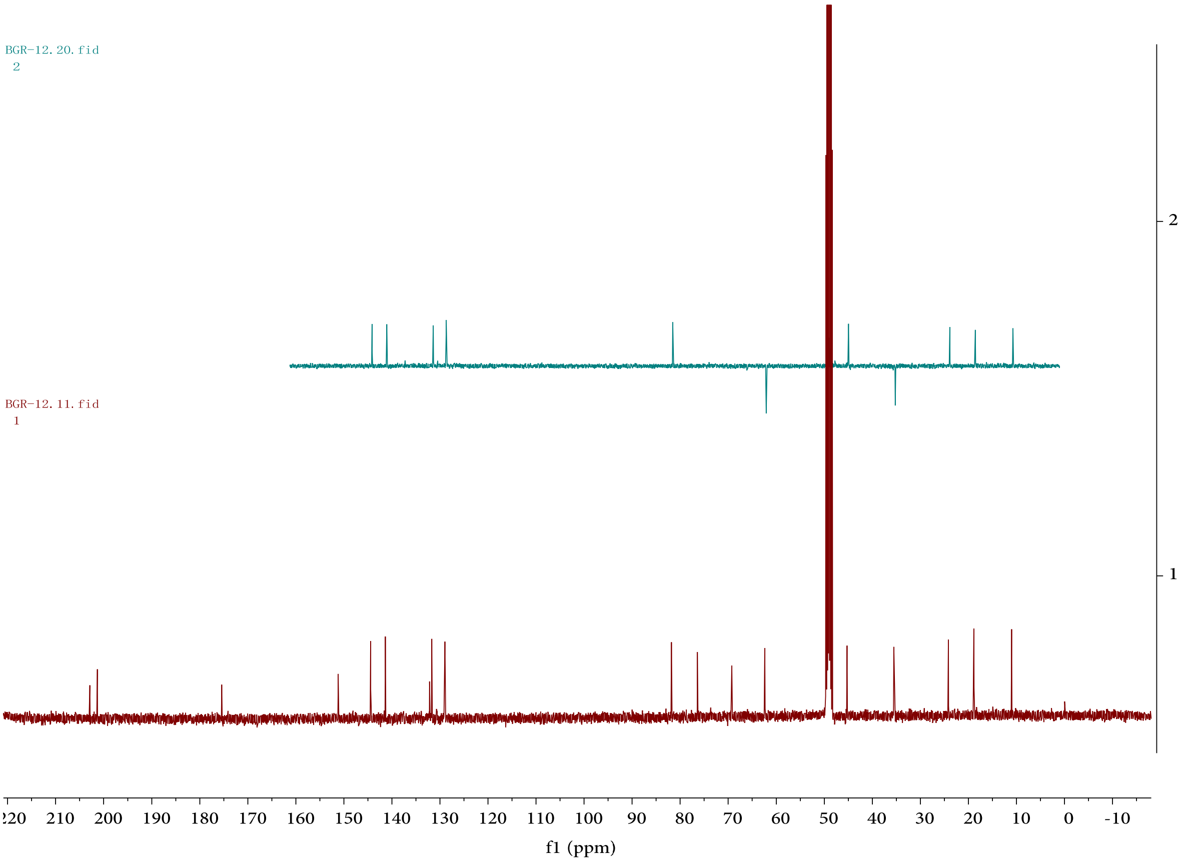

Fig.S4. DEPT135 spectrum of compound **1** (400 MHz, CD_3OD)

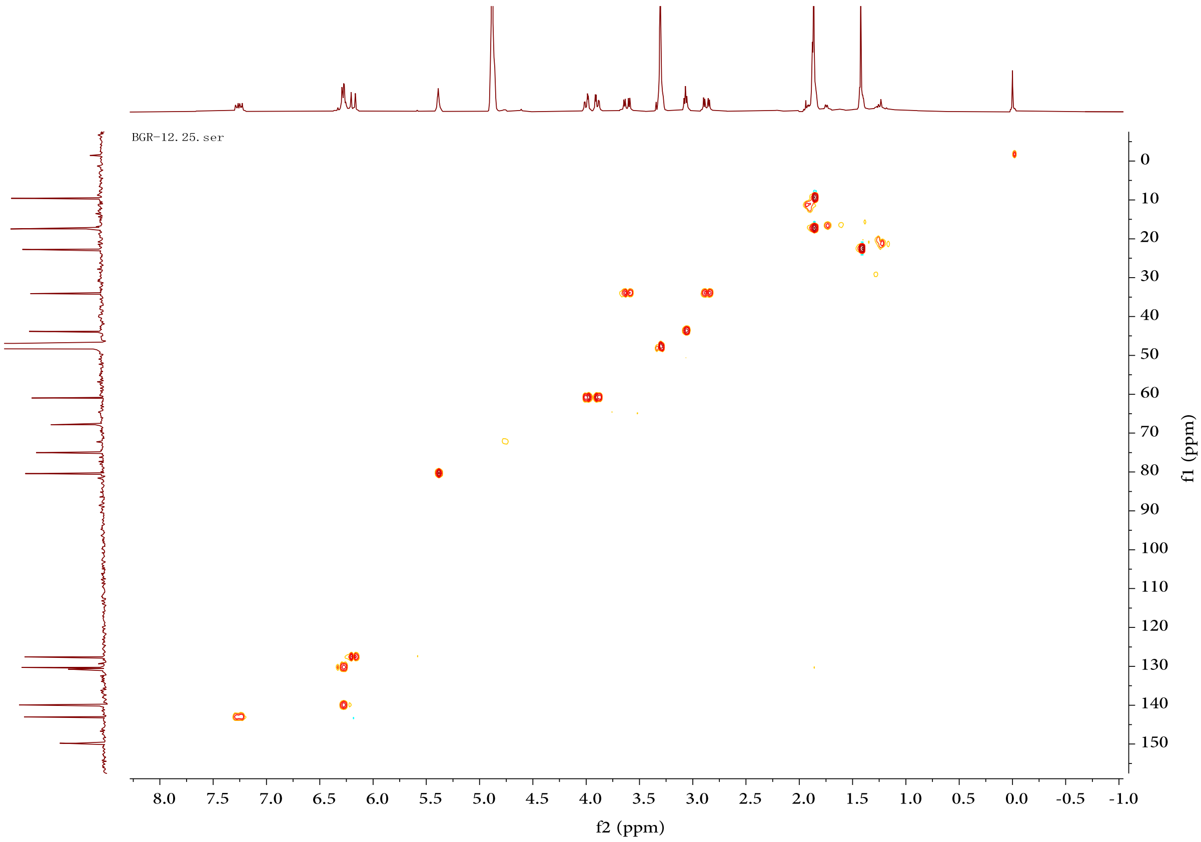

Fig.S5. HSQC spectrum of compound **1** (CD_3OD)

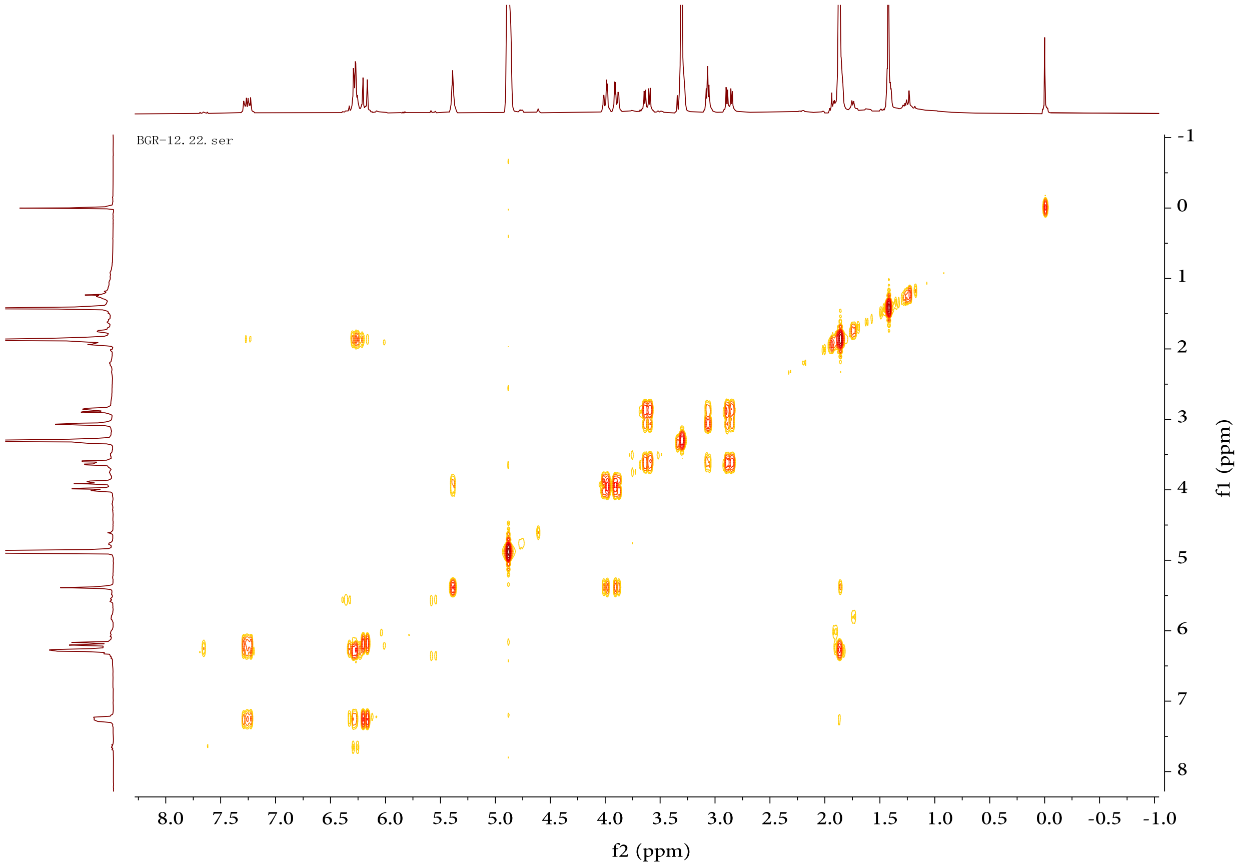

Fig.S6. H,H-COSY spectrum of compound **1** (CD_3OD)

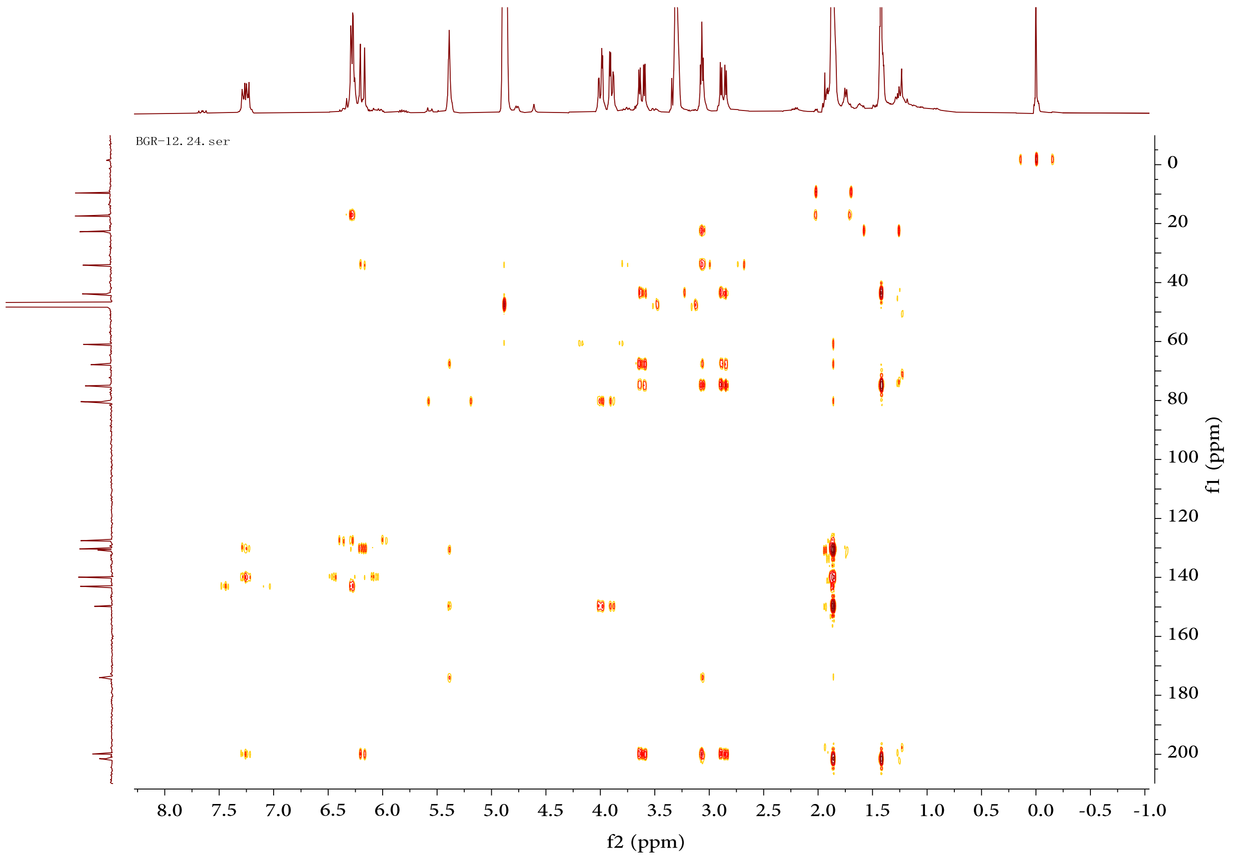

Fig.S7. HMBC spectrum of compound **1** (CD_3OD)

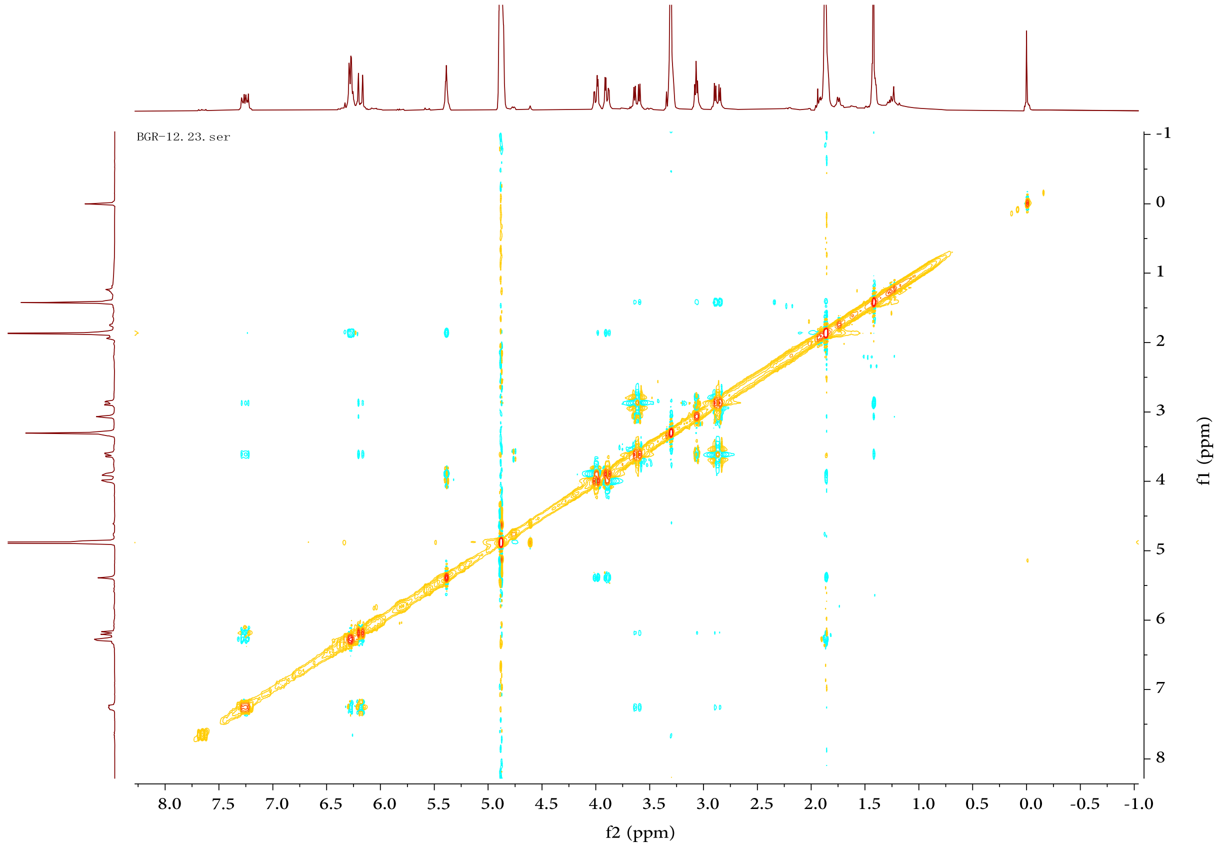

Fig.S8. NOE spectrum of compound **1** (CD_3OD)

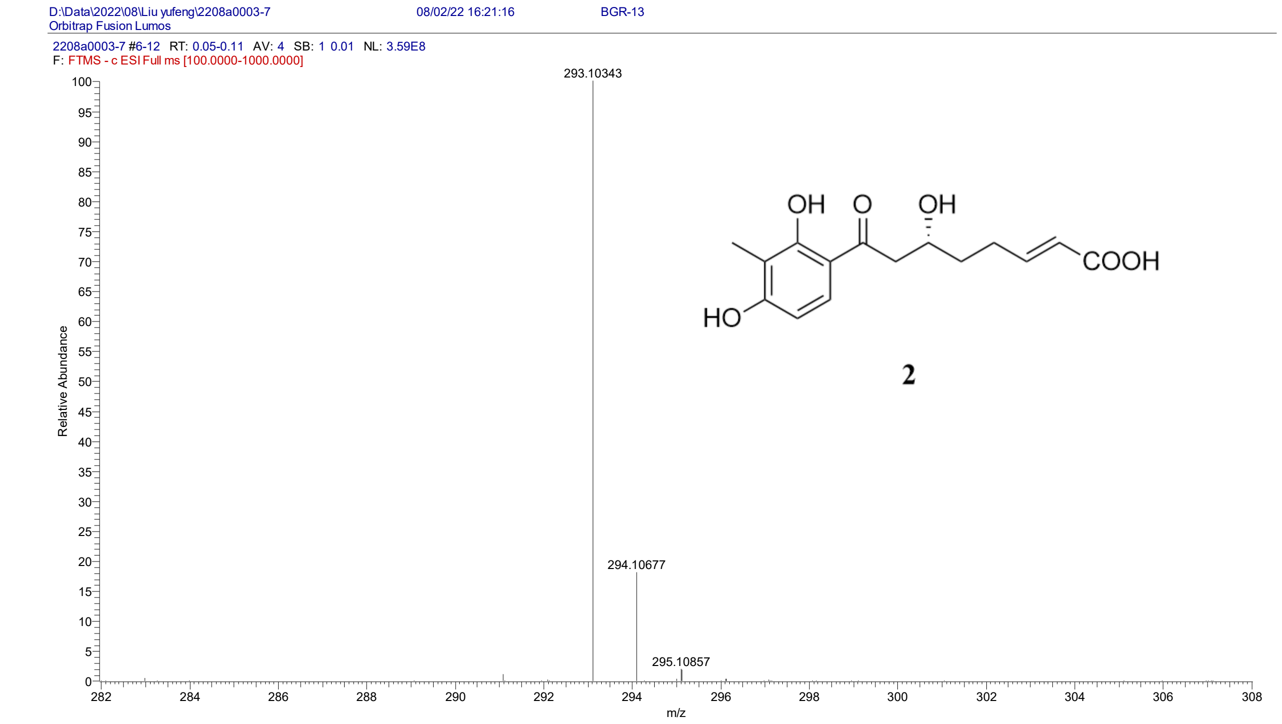

Fig.S9. HRESIMS spectrum of compound **2**

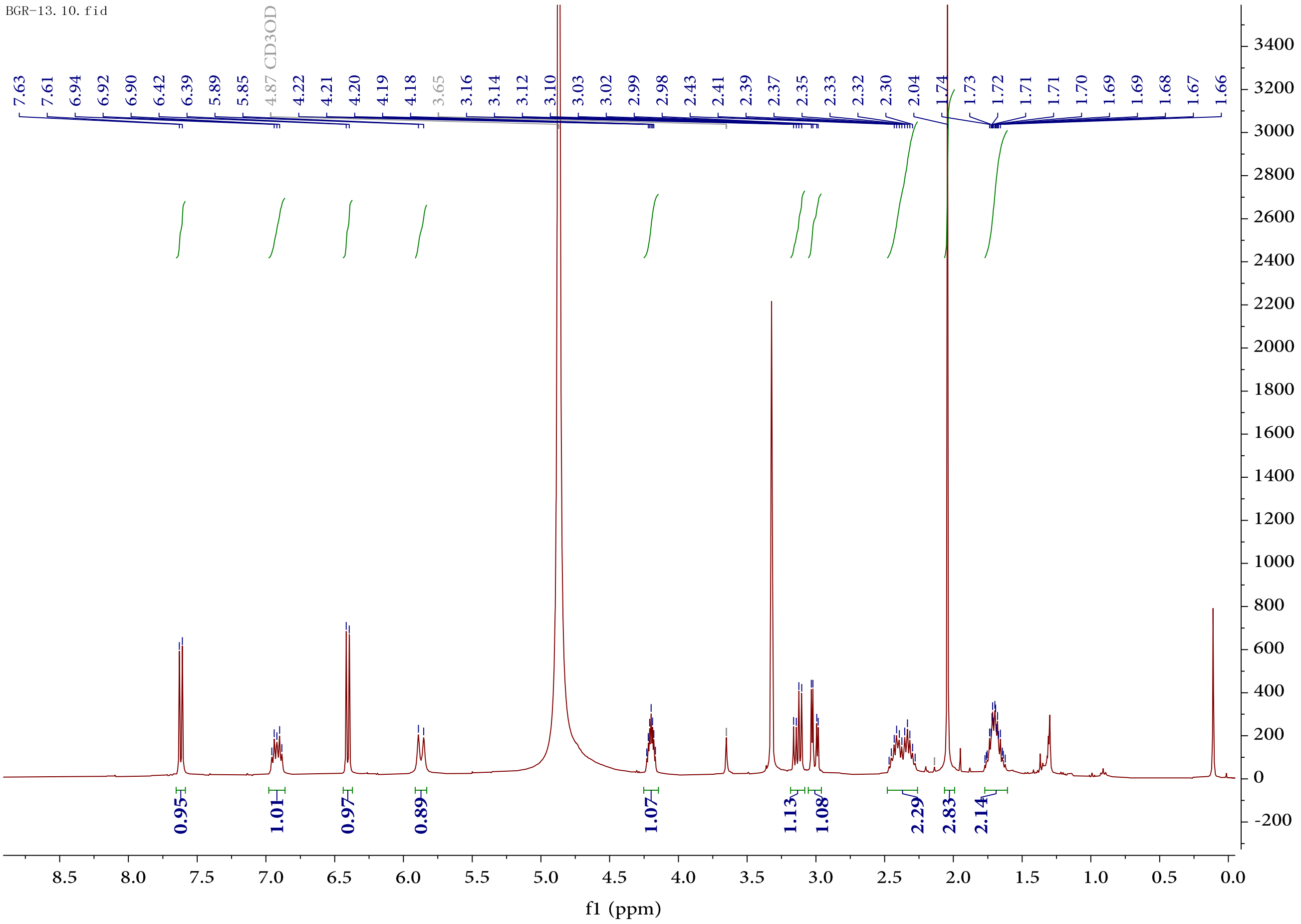

Fig.S10. ^{1}H NMR spectrum of compound **2** (400 MHz, CD_3OD)

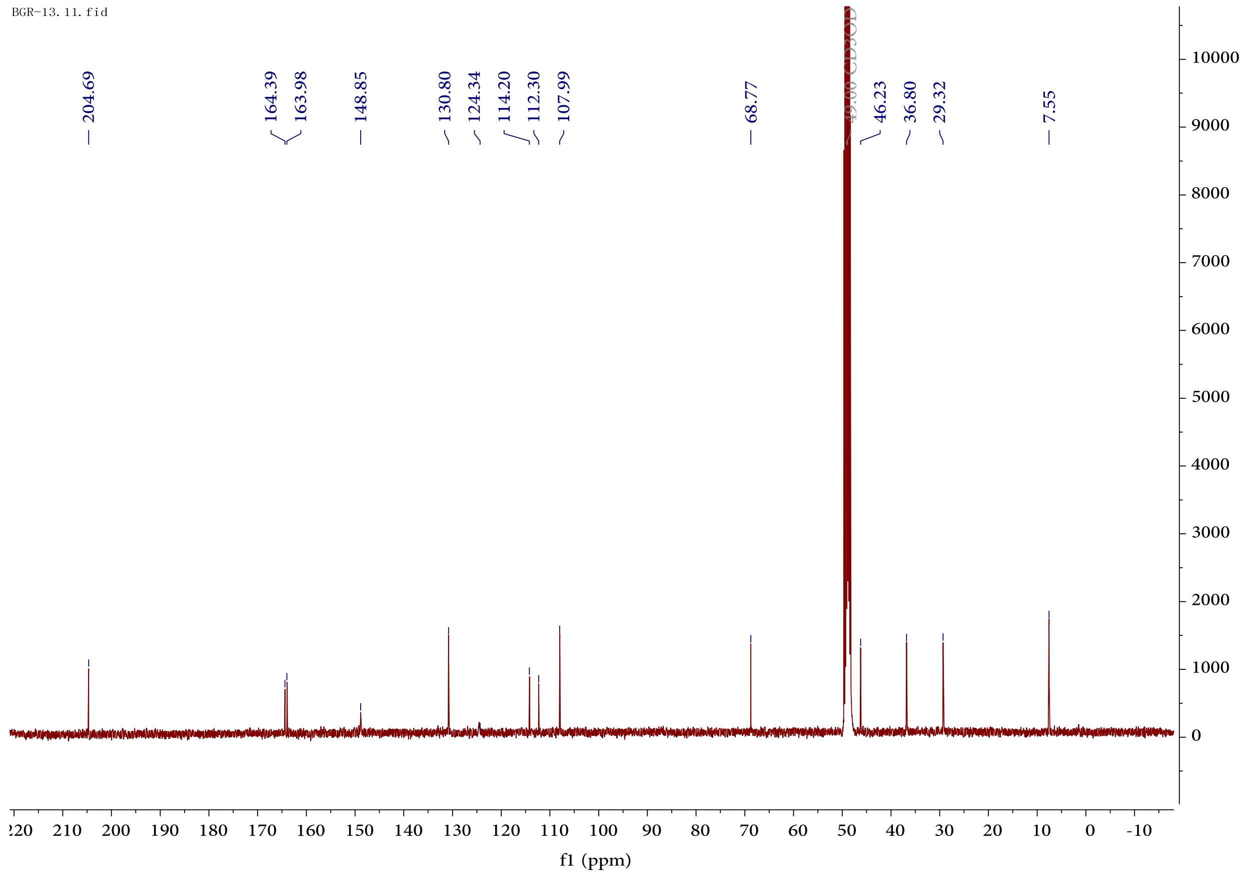

Fig.S11. ^{13}C NMR spectrum of compound **2** (400 MHz, CD_3OD)

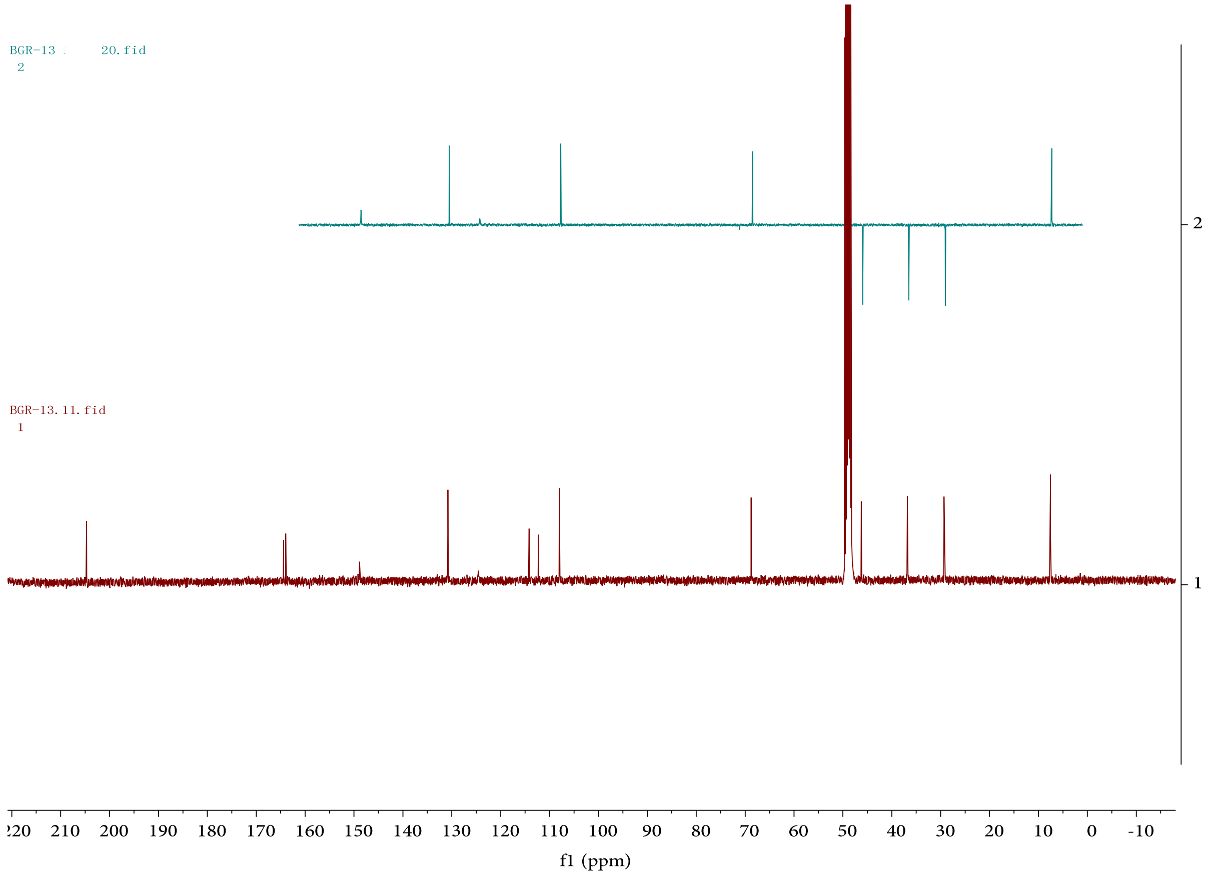

Fig.S12. DEPT135 spectrum of compound **2** (400 MHz, CD_3OD)

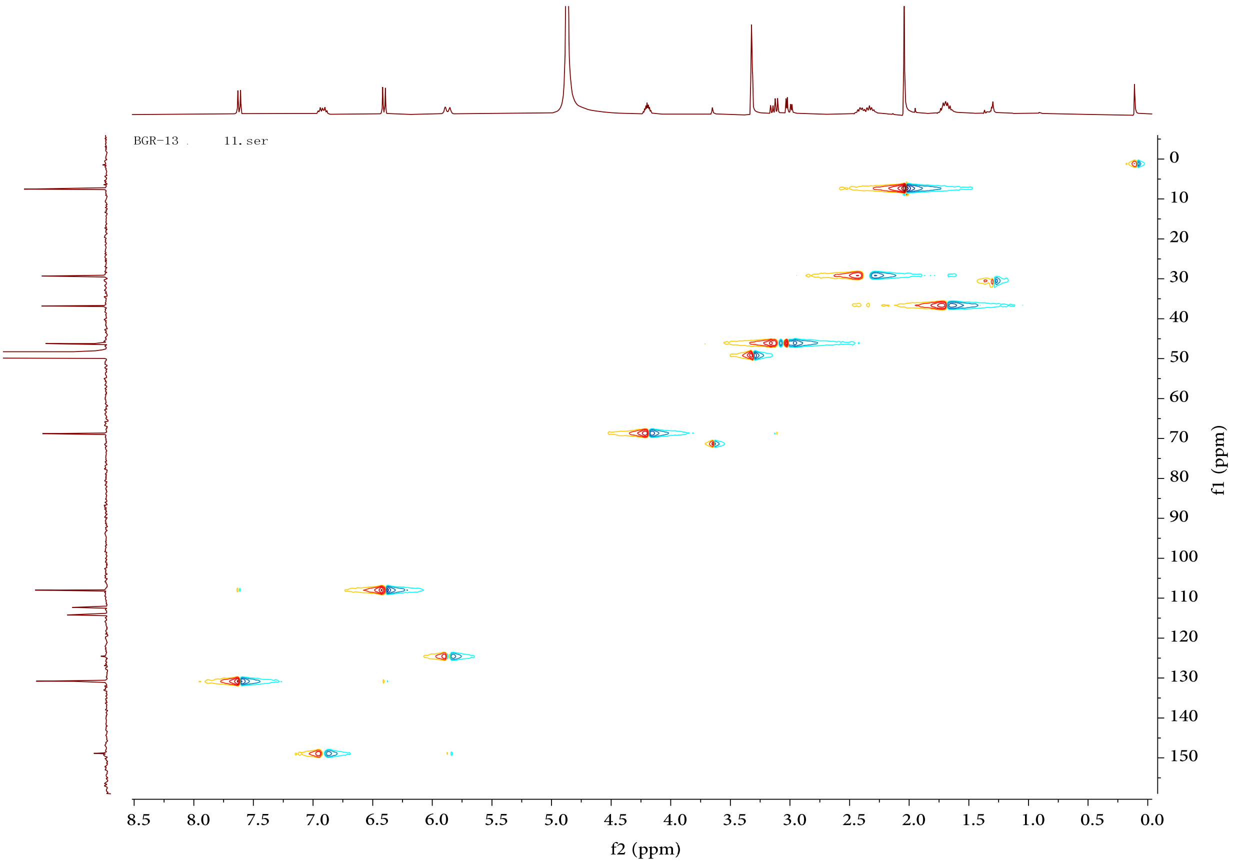

Fig.S13. HSQC spectrum of compound **2** (CD_3OD)

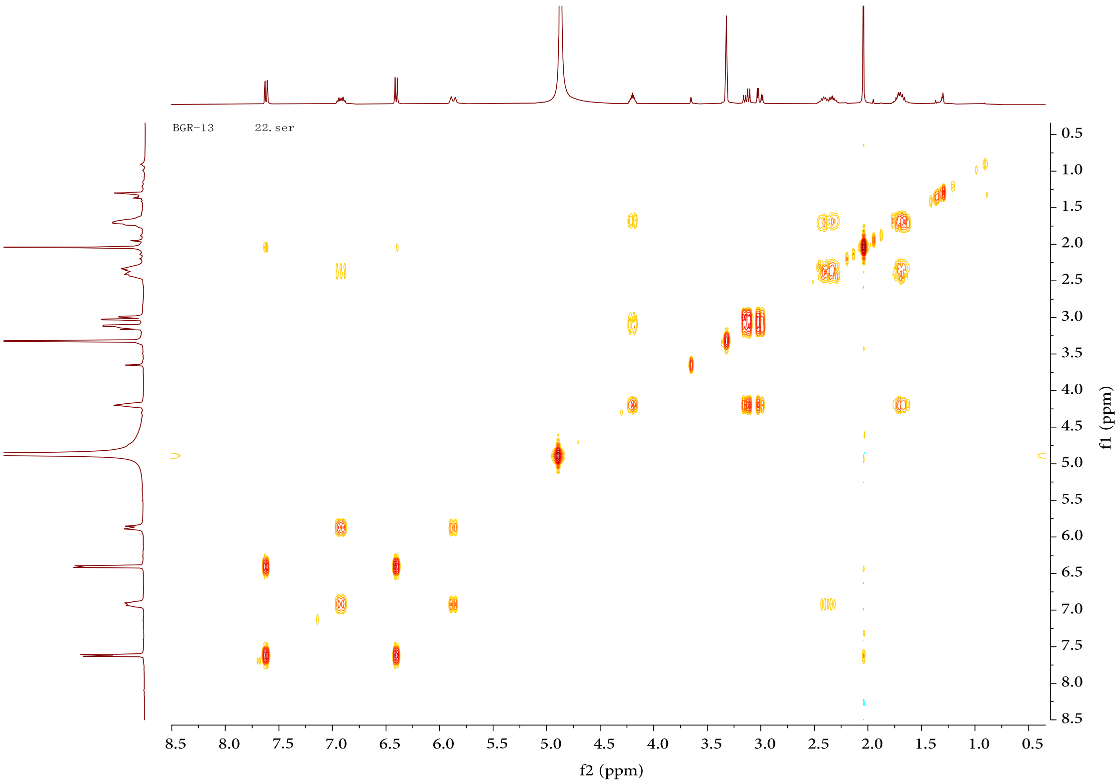

Fig.S14. H,H-COSY spectrum of compound **2** (CD_3OD)

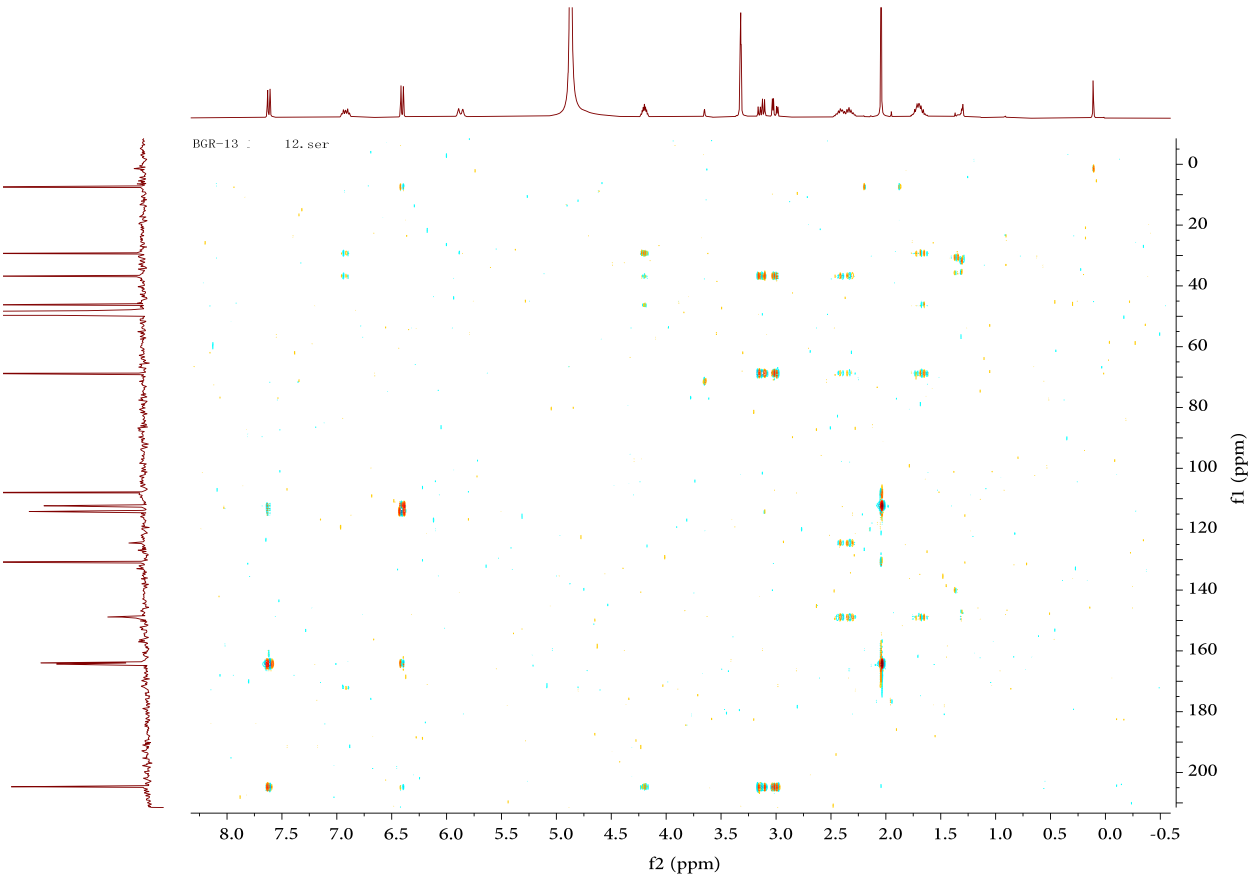

Fig.S15. HMBC spectrum of compound **2** (CD_3OD)

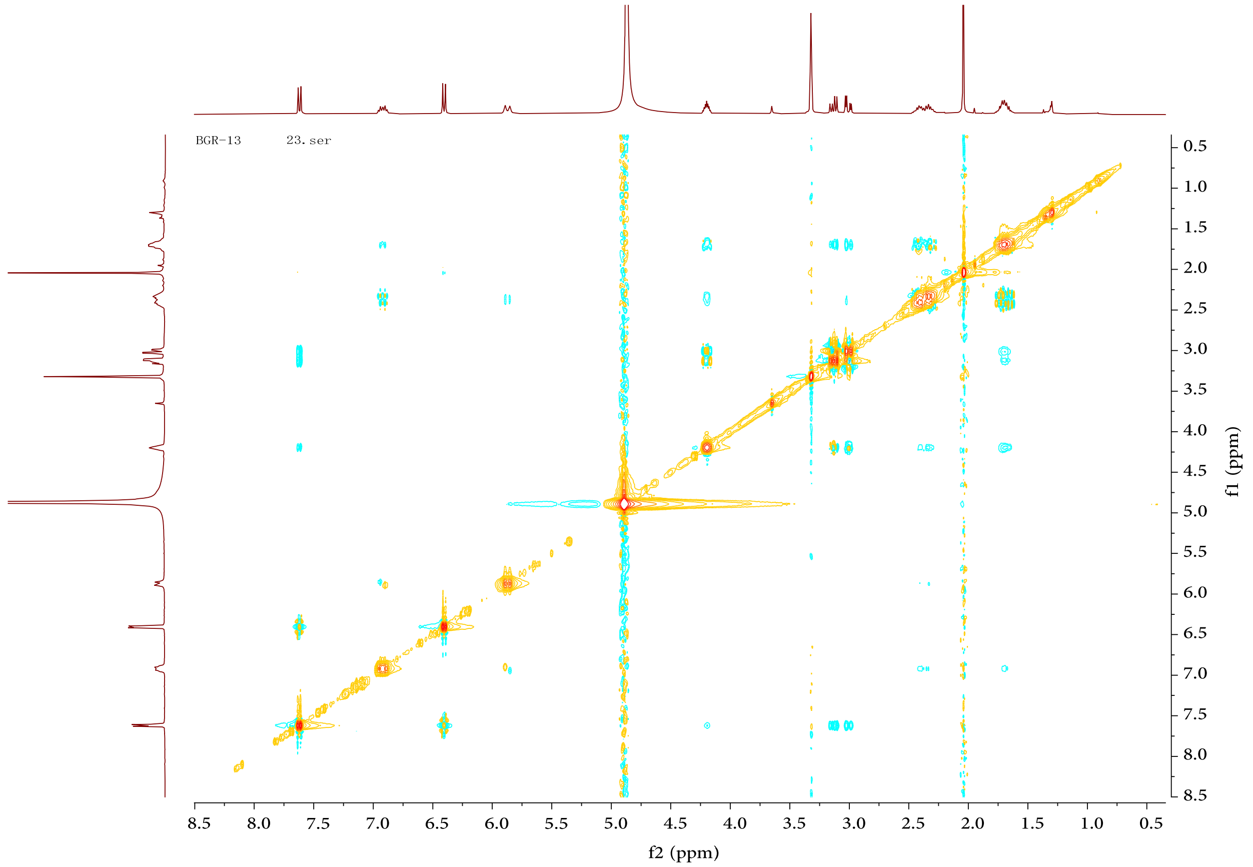

Fig.S16. NOE spectrum of compound **2** (CD_3OD)

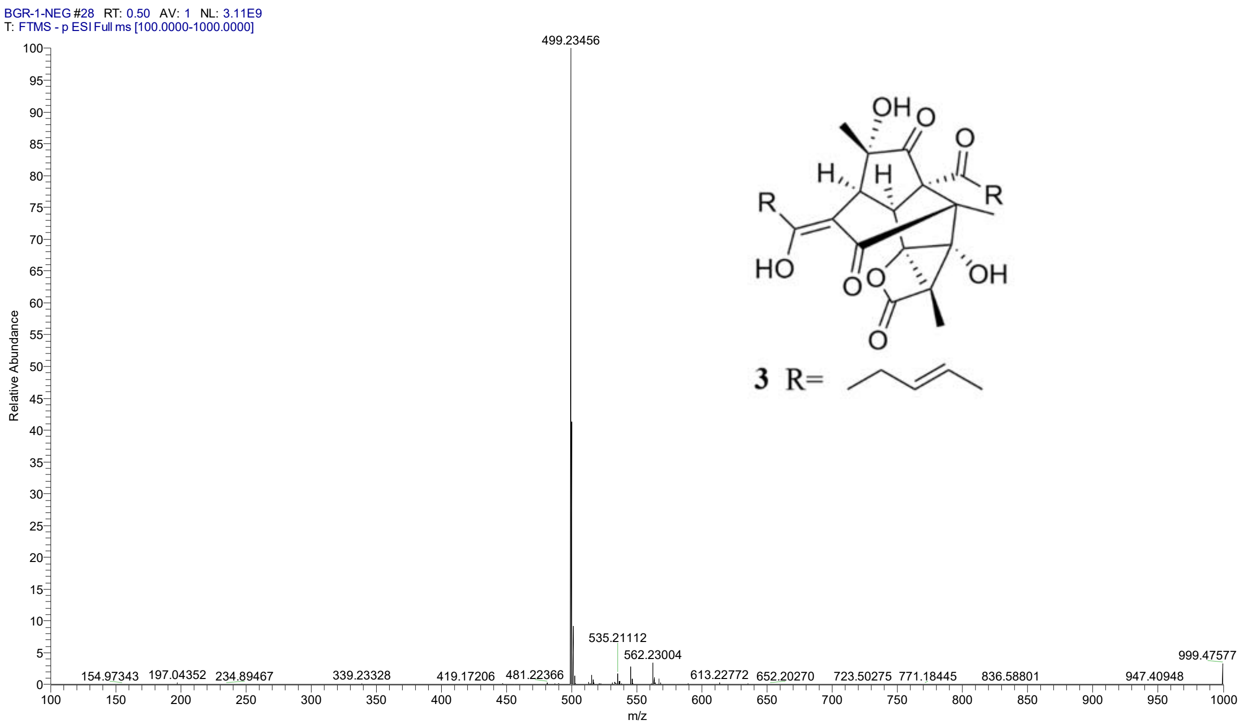

Fig.S17. HRESIMS spectrum of compound **3**

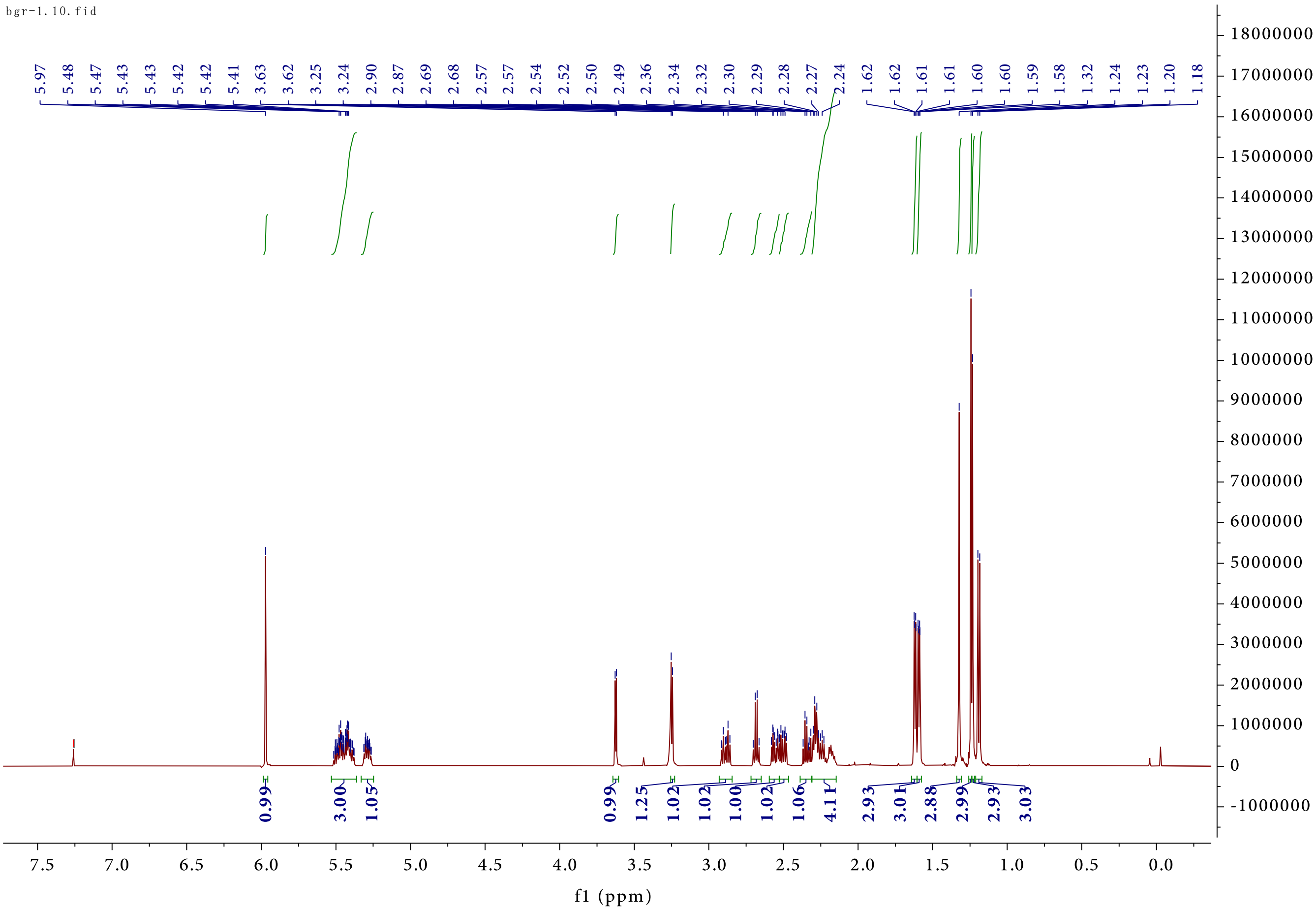

Fig.S18. ^{1}H NMR spectrum of compound **3** (400 MHz, $CDCl_3$)

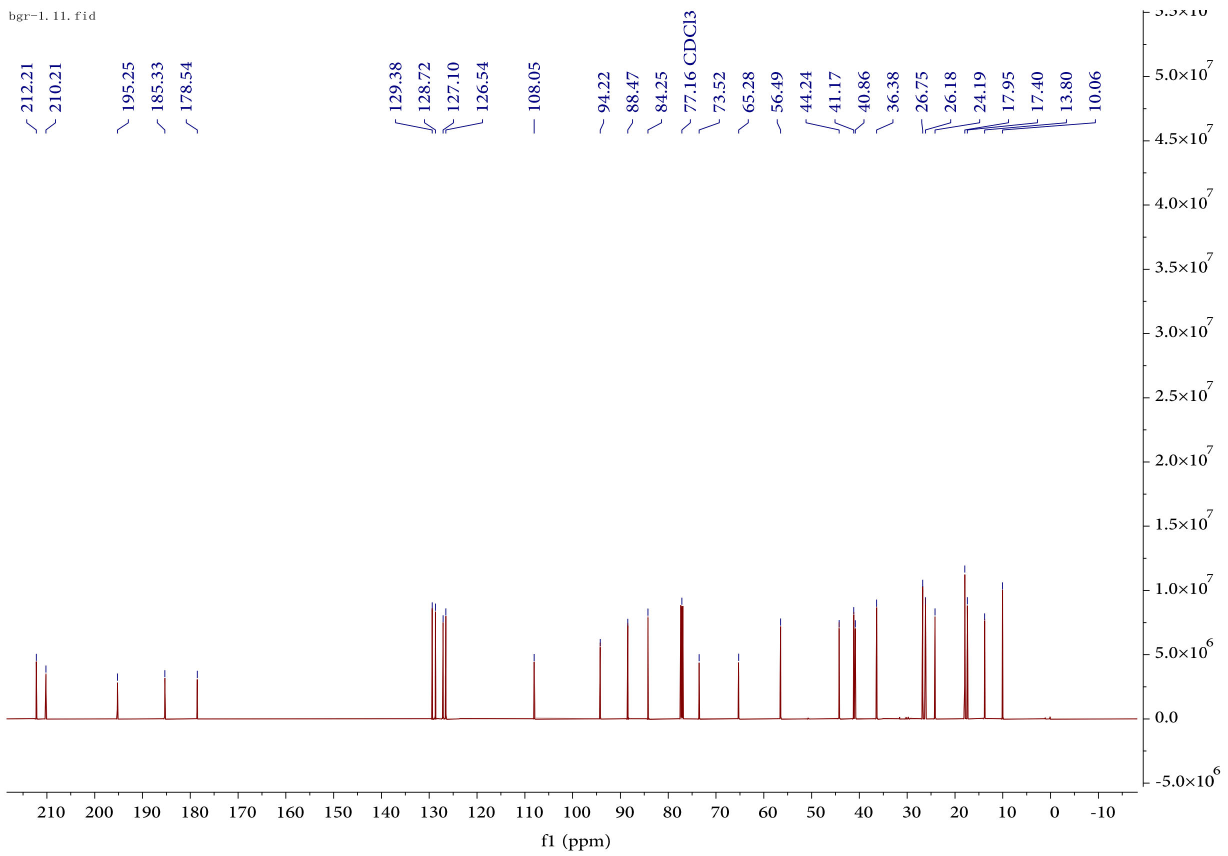

Fig.S19. ^{13}C NMR spectrum of compound **3** (400 MHz, $CDCl_3$)

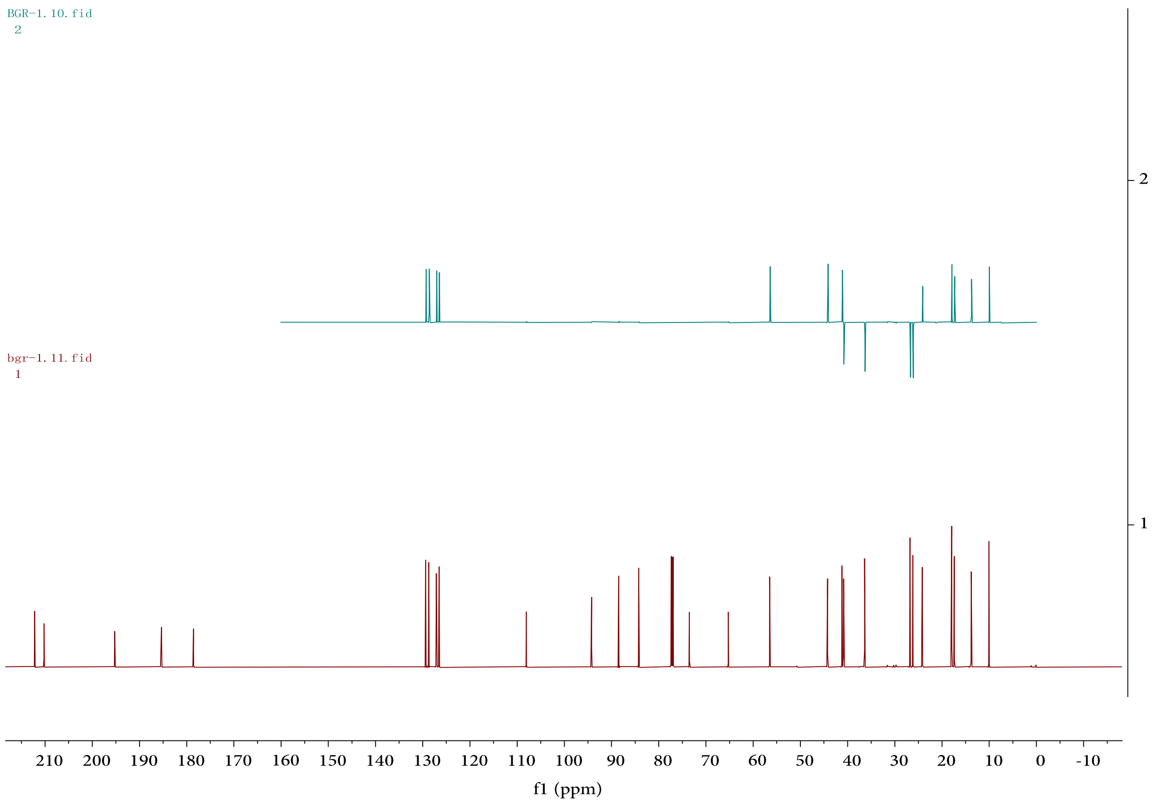

Fig.S20. DEPT135 spectrum of compound **3** (400 MHz, $CDCl_3$)

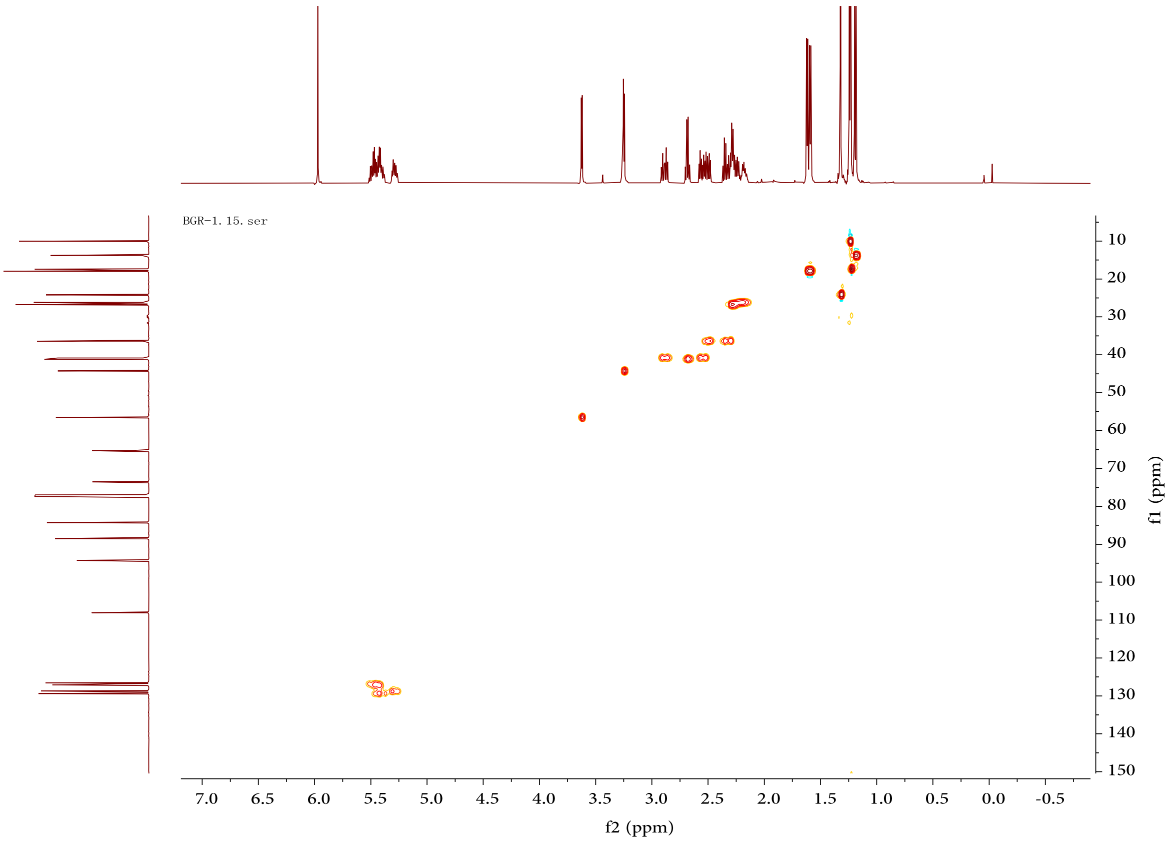

Fig.S21. HSQC spectrum of compound **3** ($CDCl_3$)

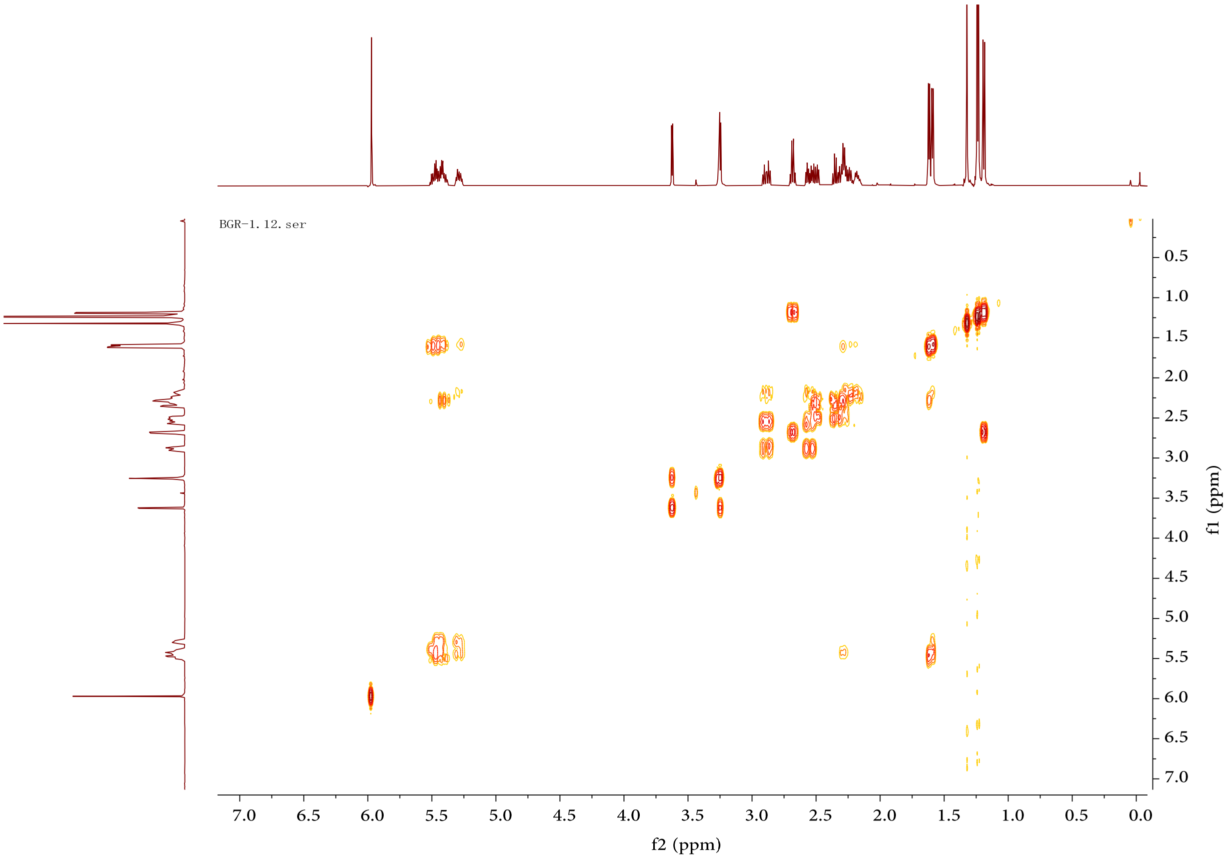

Fig.S22. H,H-COSY spectrum of compound **3** ($CDCl_3$)

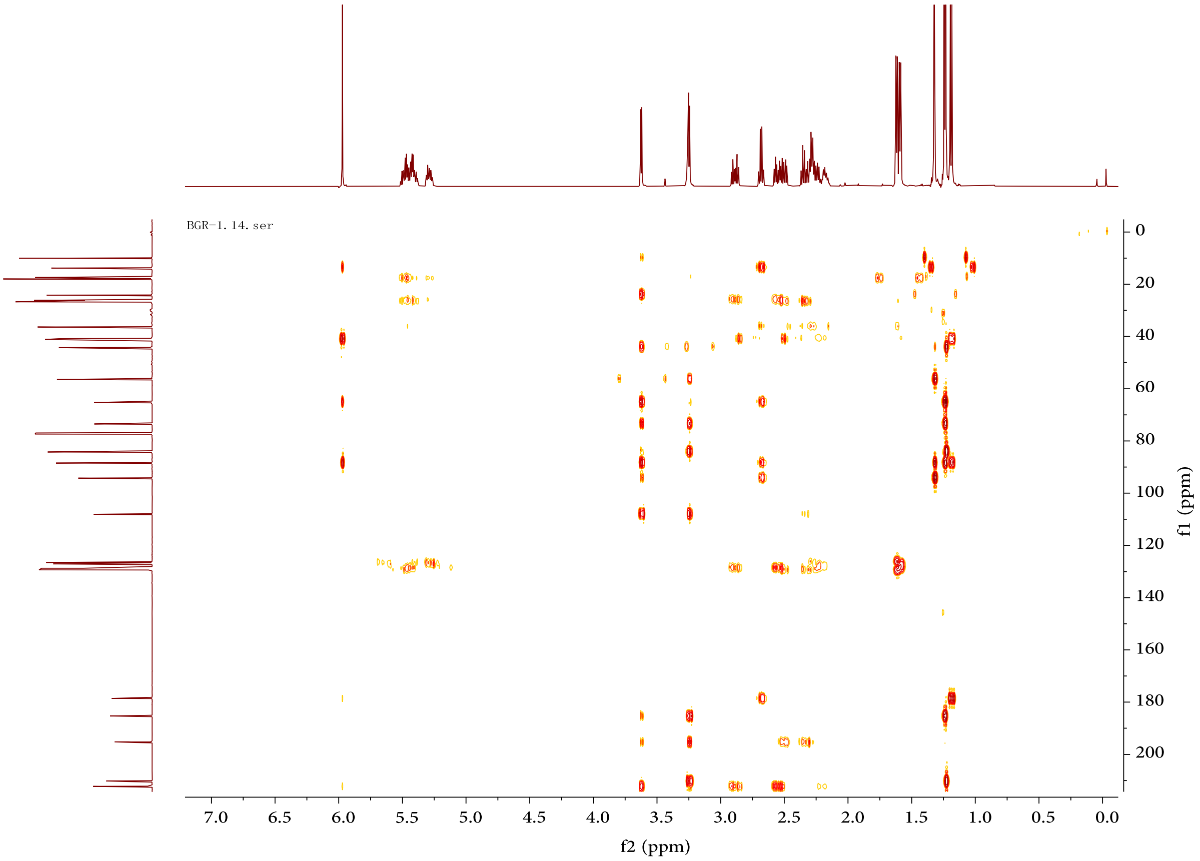

Fig.S23. HMBC spectrum of compound **3** ($CDCl_3$)

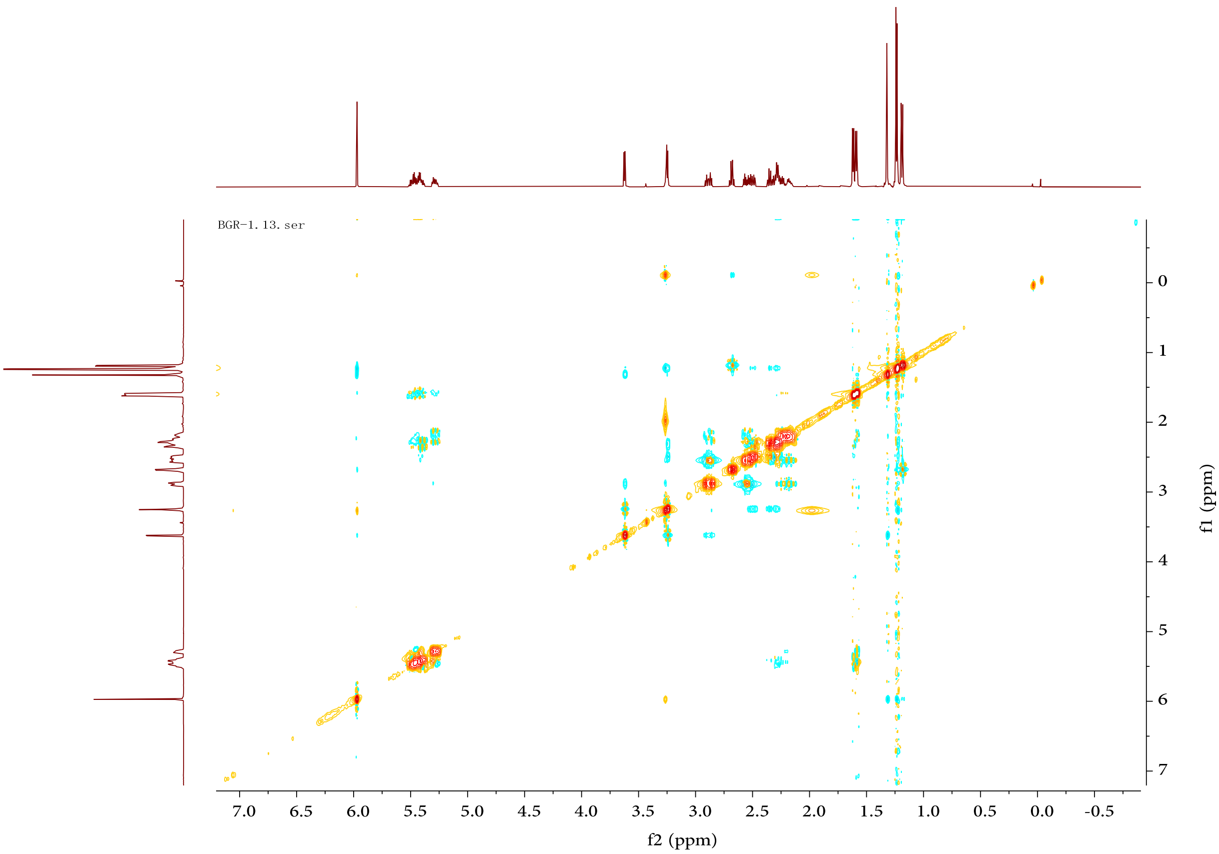

Fig.S24. NOE spectrum of compound **3** ($CDCl_3$)

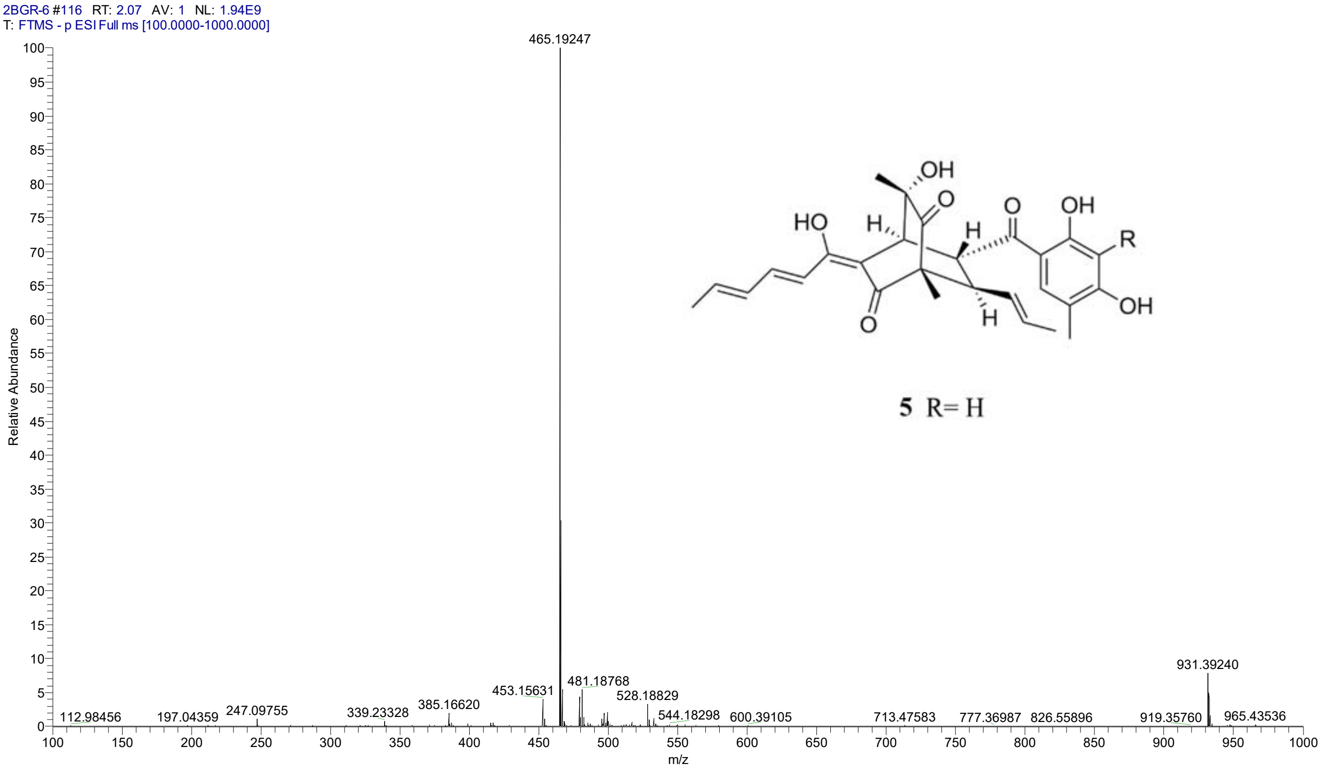

Fig.S25. HRESIMS spectrum of compound **5**

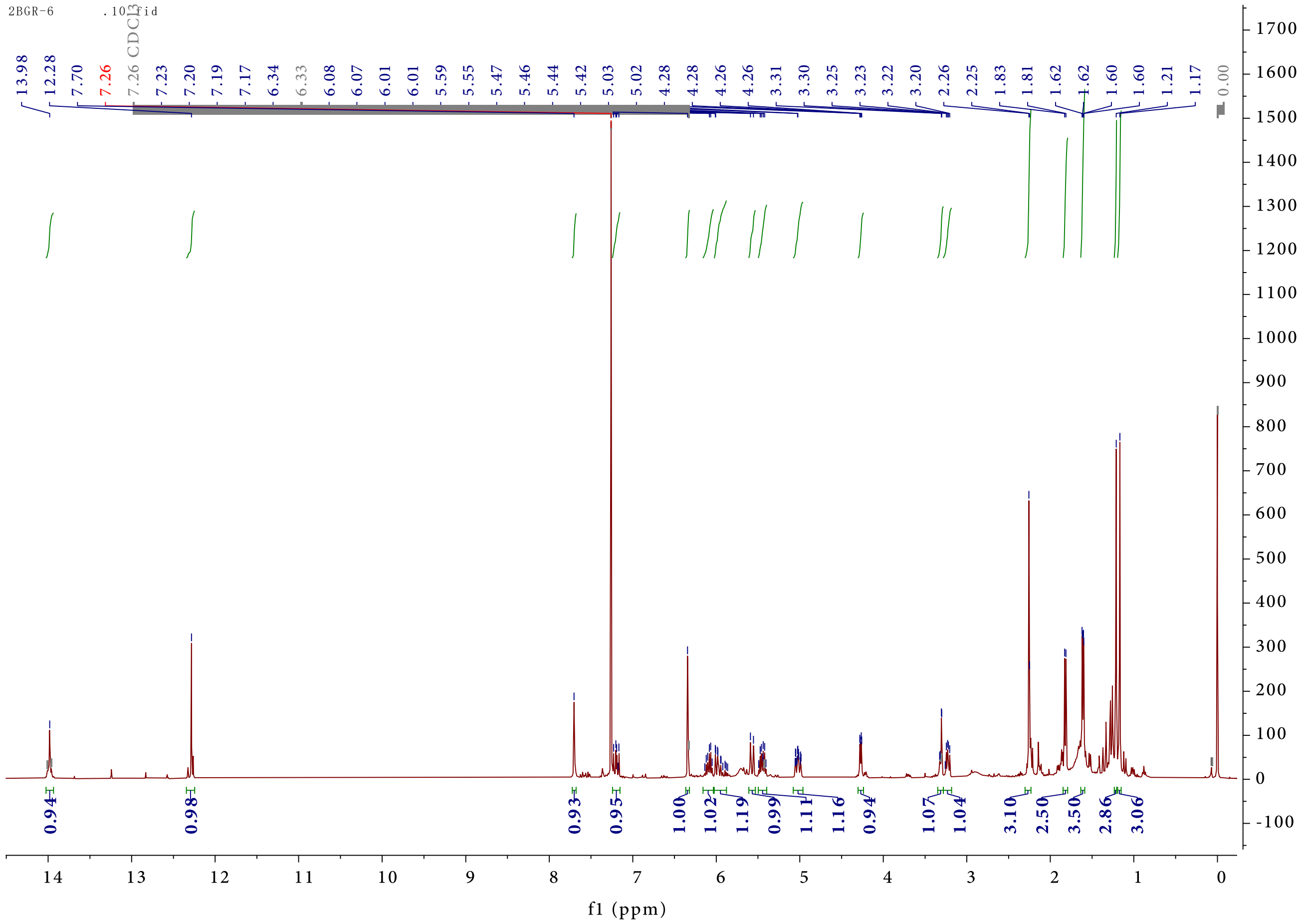

Fig.S26. ^{1}H NMR spectrum of compound **5** (400 MHz, $CDCl_3$)

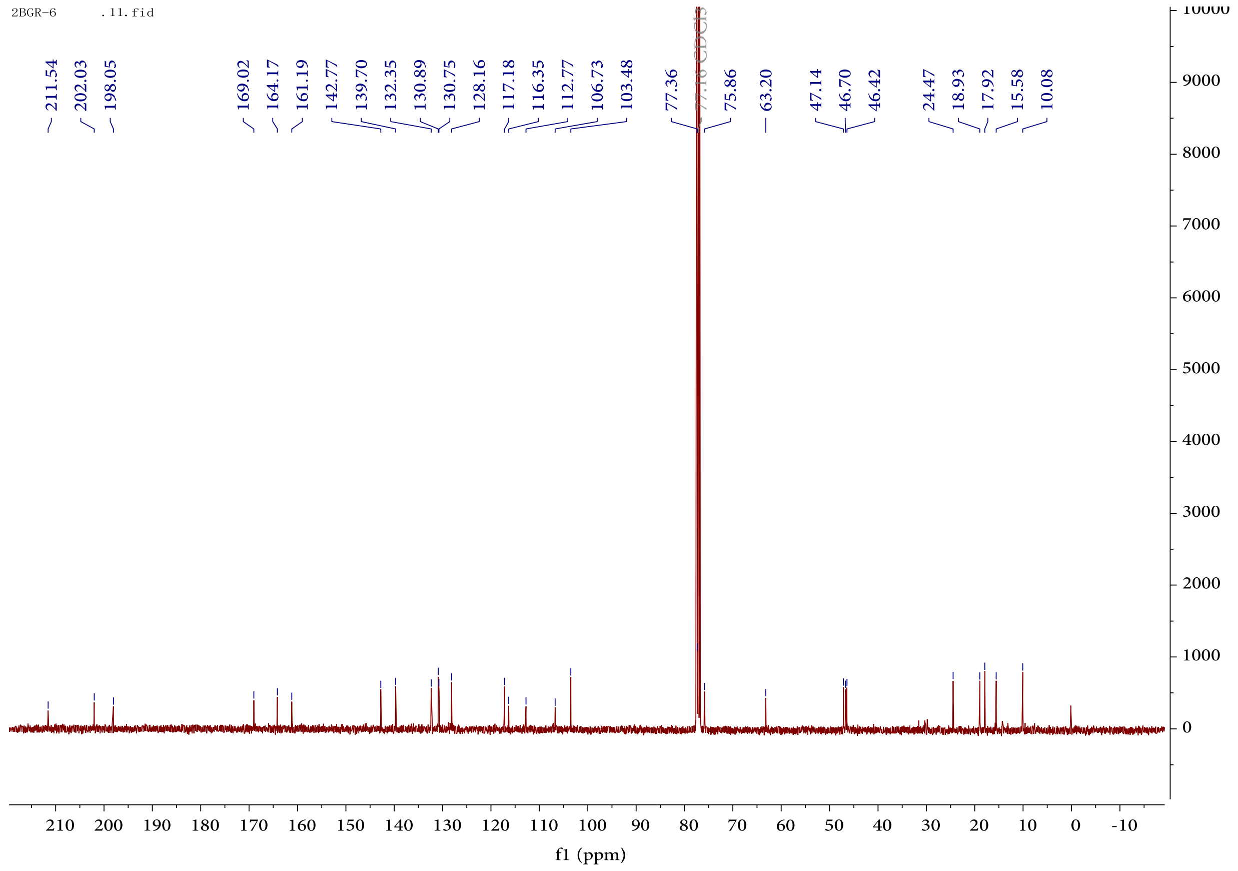

Fig.S27. ^{13}C NMR spectrum of compound **5** (400 MHz, $CDCl_3$)

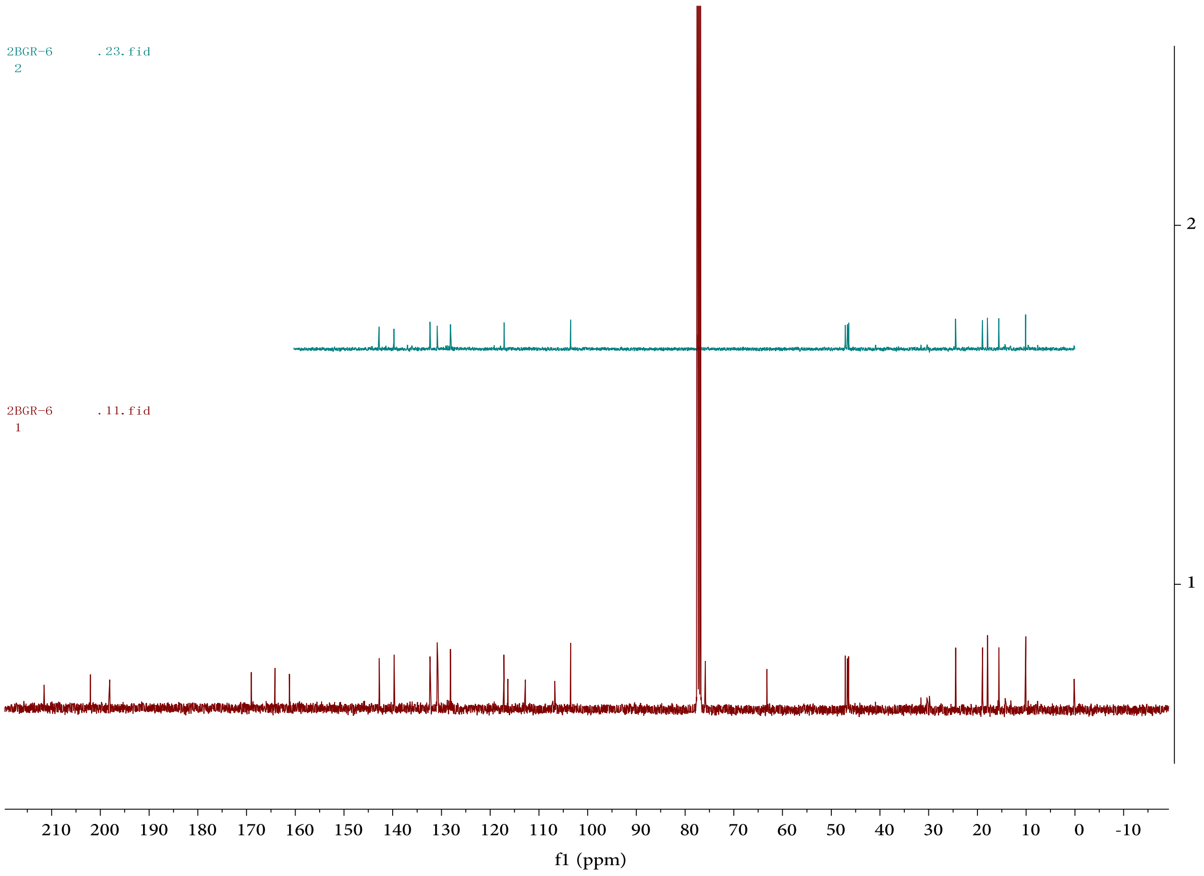

Fig.S28. DEPT135 spectrum of compound **5** (400 MHz, $CDCl_3$)

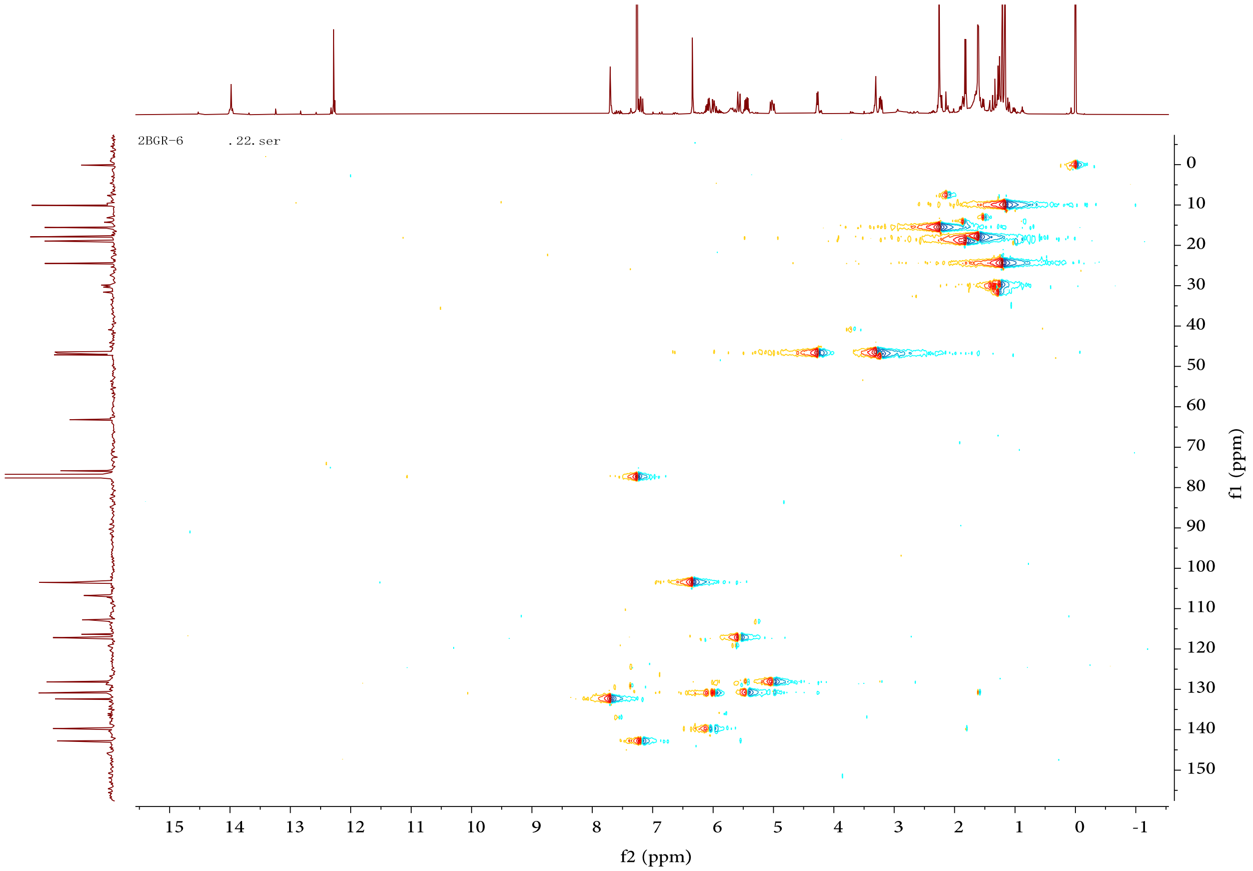

Fig.S29. HSQC spectrum of compound **5** ($CDCl_3$)

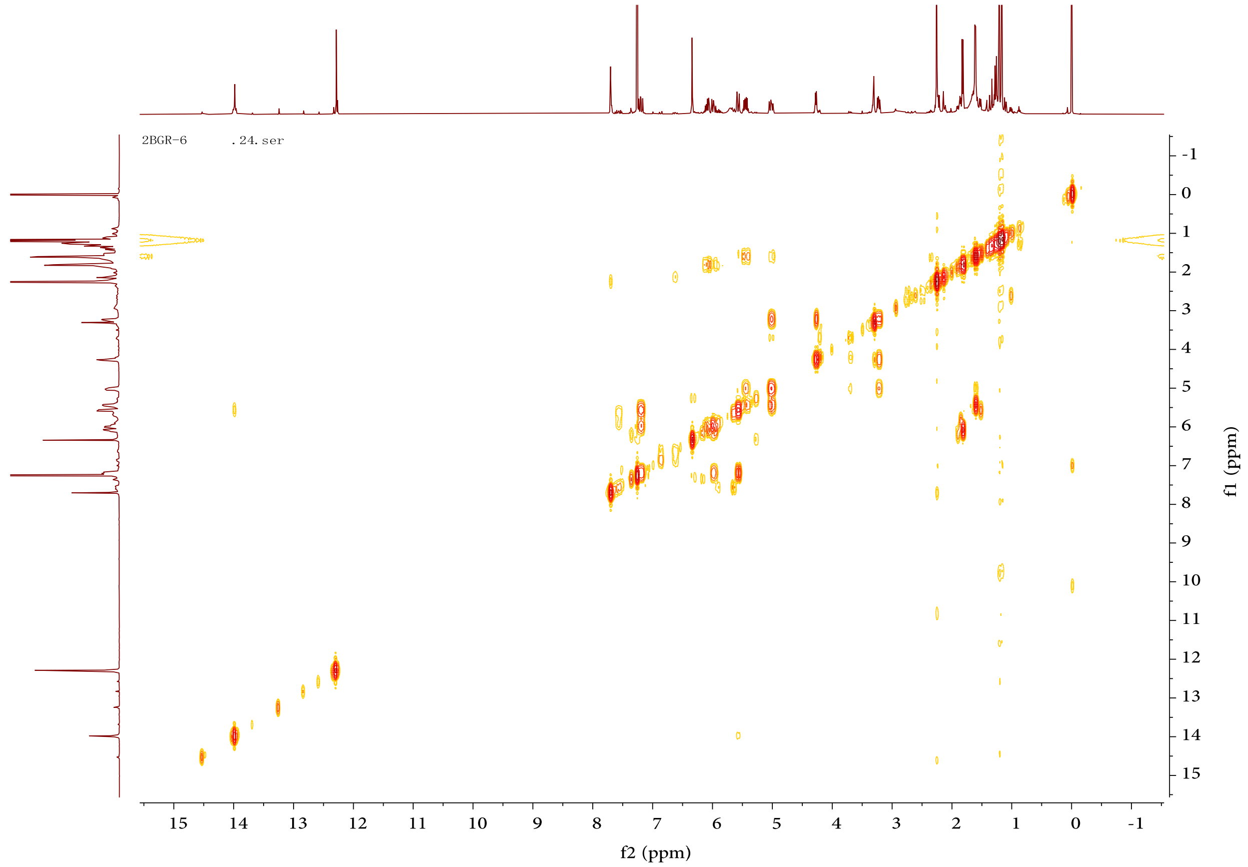

Fig.S30. H,H-COSY spectrum of compound **5** ($CDCl_3$)

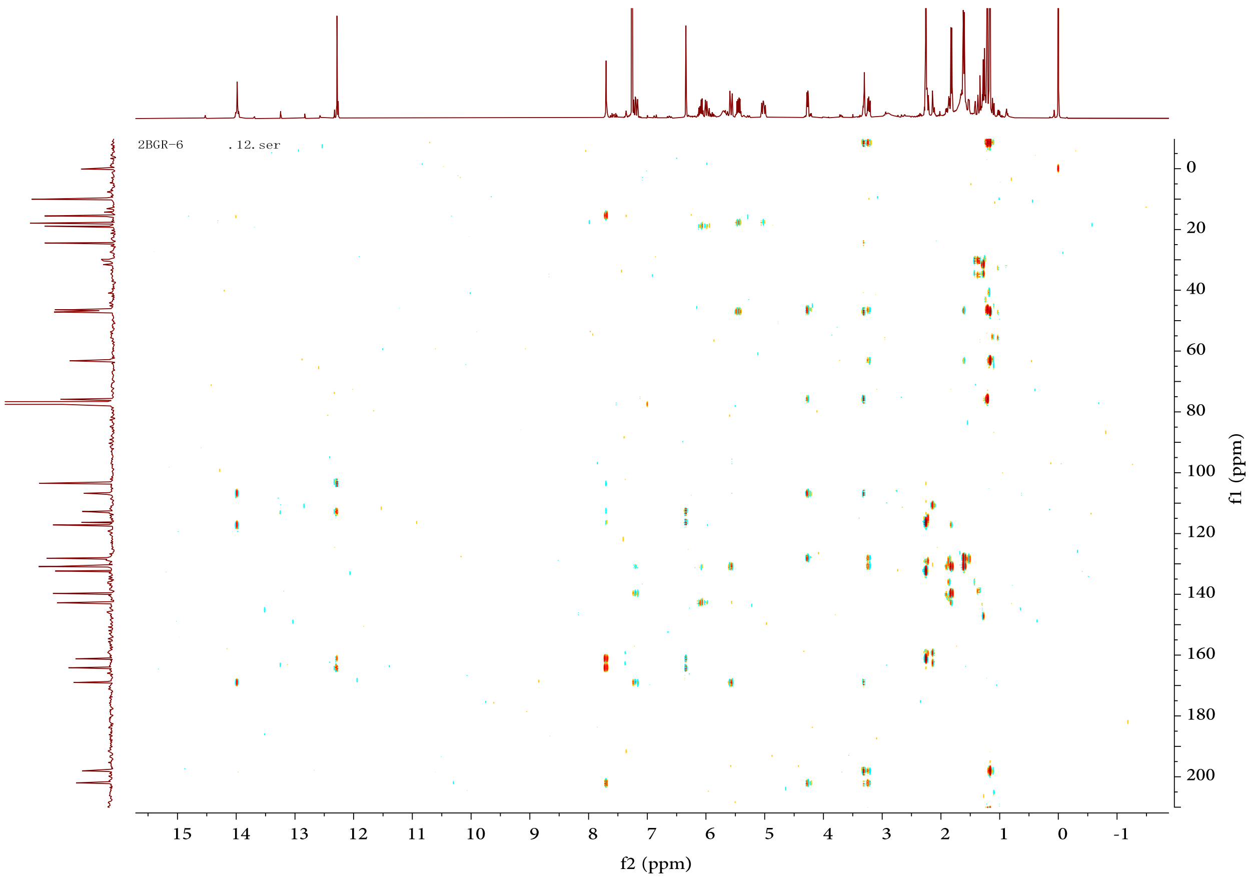

Fig.S31. HMBC spectrum of compound **5** ($CDCl_3$)

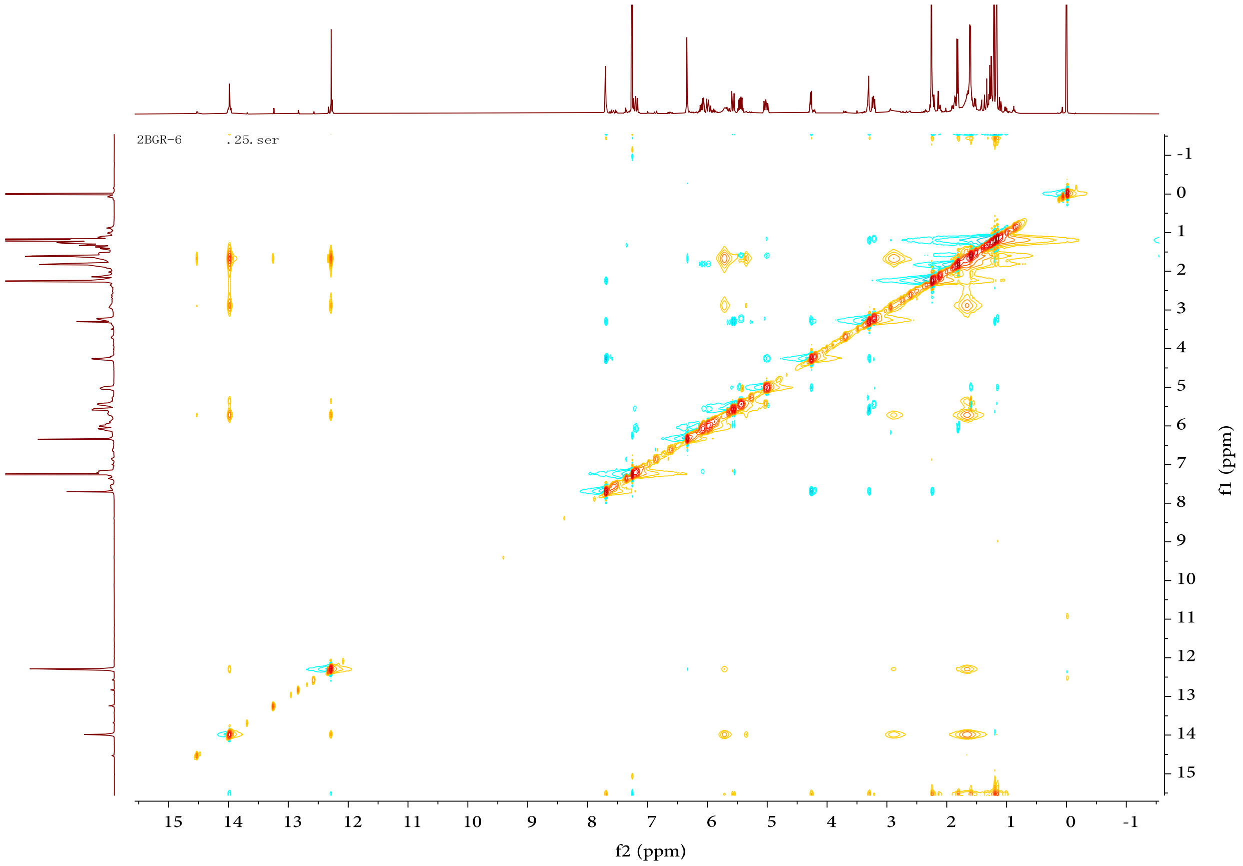

Fig.S32. NOE spectrum of compound **5** ($CDCl_3$)

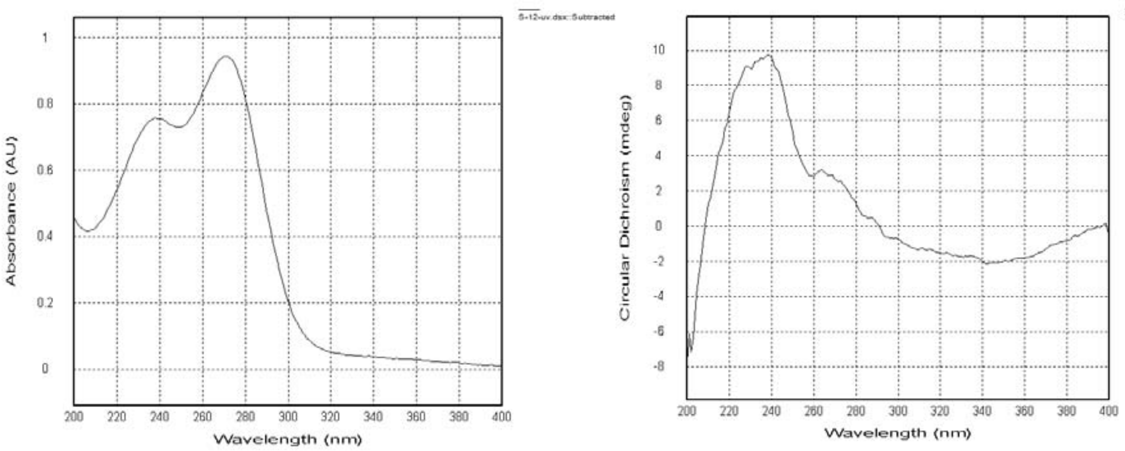

Fig.S33. UV and ECD of compound **1** (CH_3OH)

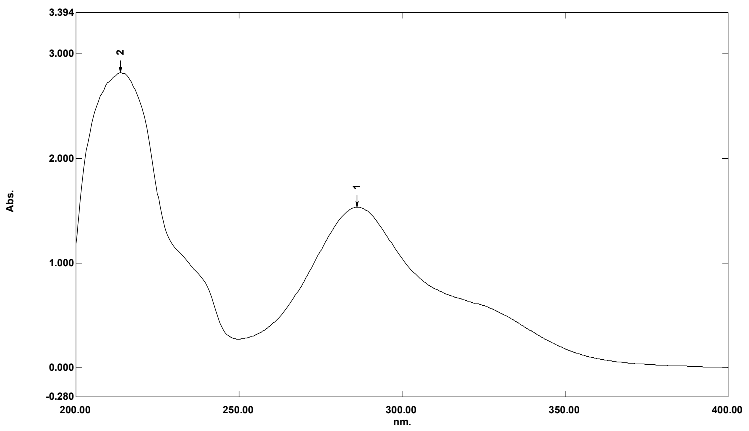

Fig.S34. UV of compound **2** (CH_3OH)

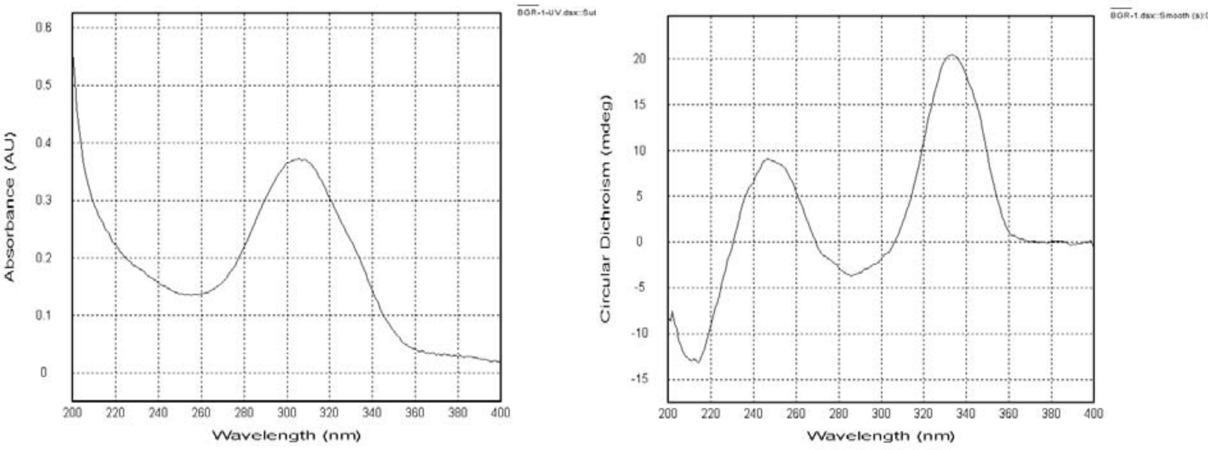

Fig.S35. UV and ECD of compound **3** (CH_3OH)

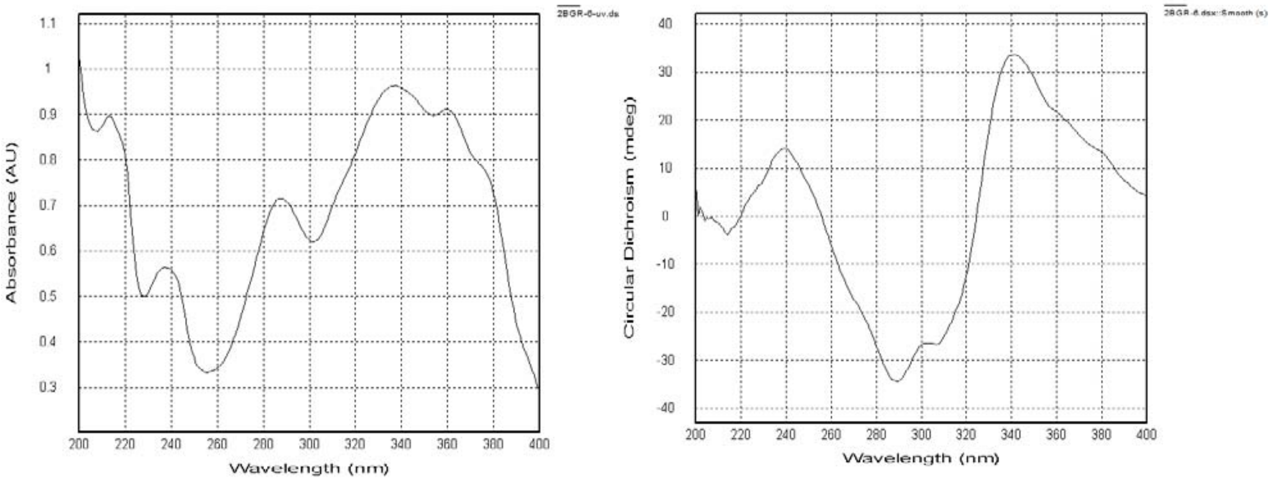

Fig.S36. UV and ECD of compound **5** (CH_3OH)

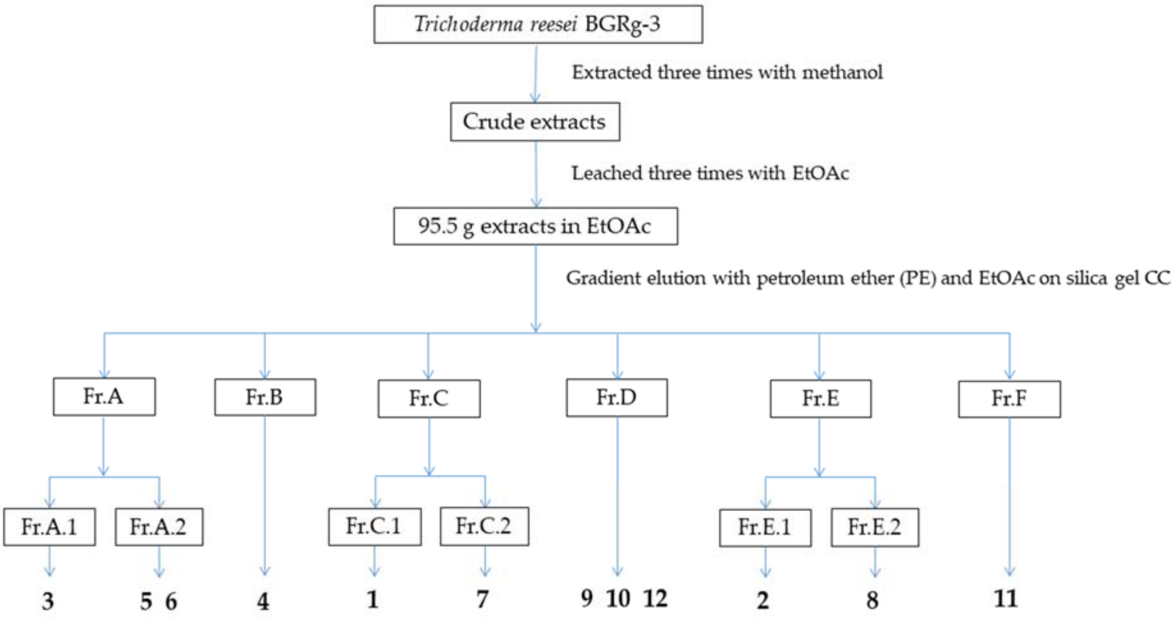

Fig.S37. Isolation procedure

Biosynthesis pathway

Biosynthesis pathways of sorbicillinoids had been explored in depth [1-3]. Recent study [2] showed that a triketide intermediate **(a)** was first synthesized through one acetyl-CoA and two malonyl-CoA **(Fig S38)**. The intermediate was then elongated by several malonyl-CoA. Generally, three malonyl-CoA were used for lengthening the carbon chains to give **(1b)**. Meanwhile, two methyl may be introduced with C-methyltransferase (C-MeT) domain of the polyketide synthase. Then intermediate **(1b)** undergone knoevenagel condensation to give the first isolatable

intermediate sorbicillin **(1c)** and other possible intermediates. These mono-sorbicillinoids, for further reactions, were stereoselectively catalyzed to give the key intermediate **(1d)** by an FAD-dependent monooxygenase (FMO, SorC) [2,3], which was then reduced to generate **11**, or performed futher reactions **(Fig S39)**. It was reported that compound **12** was possibly the premature off-loading by-product when generating **(1b)**. Therefore, it was assumed that only 2 malonyl-CoA were added to the carbon chains, leading to the production of intermediate **(2b)**. Keto-enol tautomerism, annulation and oxidation were performed to generate **12**. Moreover, elongated by four malonyl-CoA from **(a)**, intermediate **(3b)** then proceed knoevenagel condensation to give **(3c)**, which finally generated compound **2**.

Diels-Alder reaction and Michael reaction were significant pathways in the biosynthesis of dimeric sorbicillinoids [1,2]. Base on the key intermediate **(1d)**, DA reactions were proceed with different dienophiles, generating bisorbicillinol, compounds **5** and **6**. Bisorbicillinol further performed intramolecular rearrangements and Michael-addition-like reactions to give compounds **3**, **4**, **8** and their analogues. Intermediate **(1d)** could also perform intermolecular Michael dimerization reactions to generate compounds **9** and **10**.

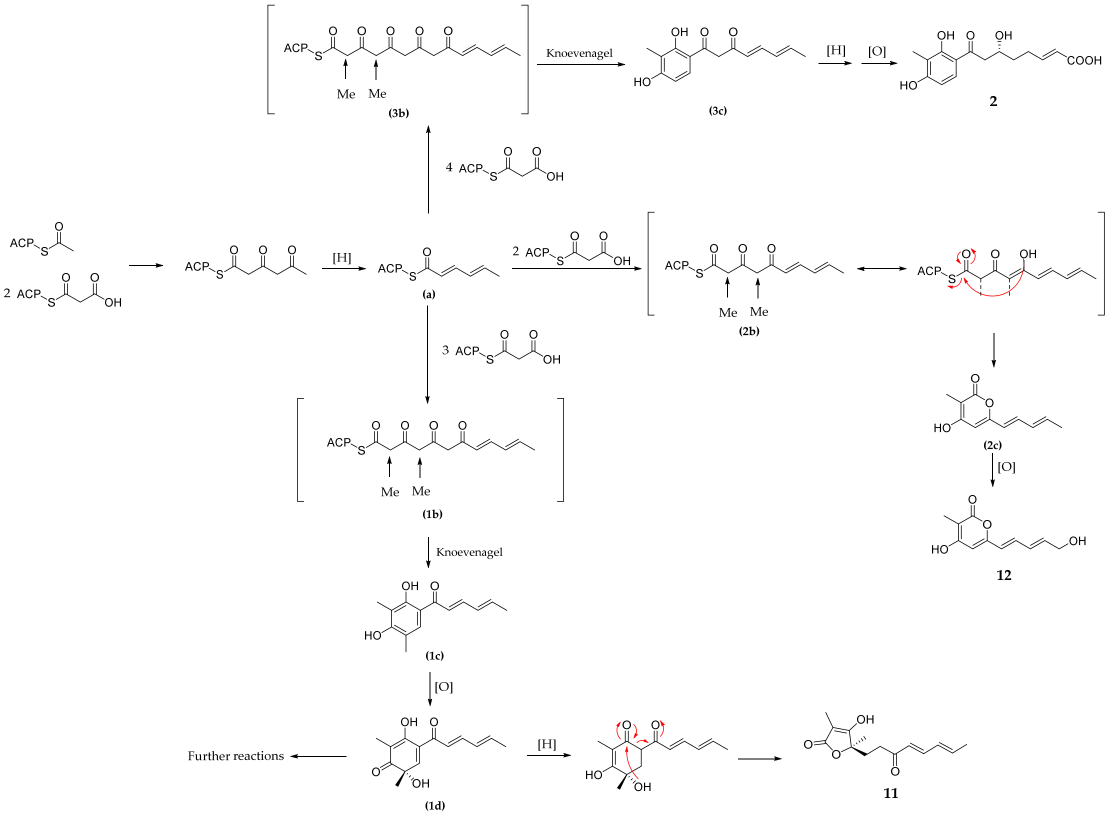

Fig.S38. Biosynthesis pathway of isolated mono-sorbicillinoids

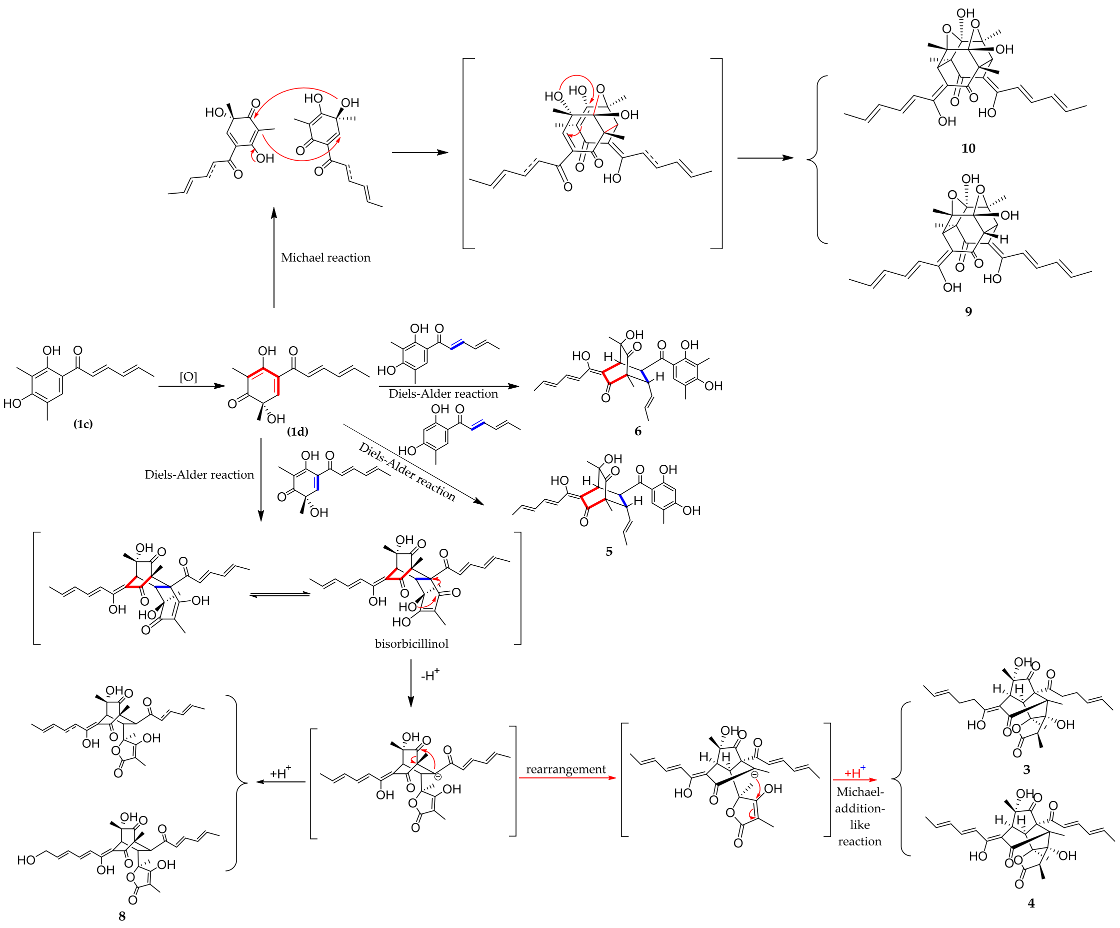

Fig.S39. Biosynthesis pathway of isolated dimeric sorbicillinoids

ECD calculations data of **1**

Conformation 1 of compound **1** (Boltzmann distribution: 0.425)

Atom	x	y	z
O	0.73379400	0.65194200	1.91618200
C	1.06066500	-0.58500300	1.51789600
C	1.16091800	-0.59702300	-0.02829800
C	2.60483100	-0.16210800	-0.36035600
C	2.97894100	1.12422800	-0.12741800
C	1.97022800	2.08101600	0.41196300
C	0.55716000	1.53169700	0.72230200
O	1.25960400	-1.50384100	2.28732100
C	4.34066800	1.72502300	-0.36043900
O	2.23969700	3.25871500	0.60804400
C	-0.42557400	2.61594500	1.09748200
C	0.16926500	0.53379300	-0.39508900
C	-1.29439400	0.08197700	-0.39833100

C	-2.21573500	1.00811500	-1.19379400
C	-3.66851600	0.83961100	-1.09845200
C	-4.31306500	-0.08541700	-0.34303100
C	-5.75152500	-0.22825300	-0.29105300
H	0.41361200	0.95179500	-1.37388900
O	-1.74358400	1.87095800	-1.94287600
C	-6.36885600	-1.16010100	0.46705500
C	-7.84820000	-1.35343400	0.56079200
O	0.79225700	-1.83176800	-0.59088800
C	3.92829600	-2.36217700	0.02249700
C	3.53798700	-1.21982700	-0.93983400
O	3.03810500	-1.70273900	-2.19416800
O	2.82928500	-3.13992000	0.53047800
H	4.77286200	2.05561400	0.59038300
H	5.04116500	1.03607900	-0.82894300
H	4.25317500	2.61419200	-0.99188800
H	-1.37703600	2.18081700	1.40964400
H	-0.02637500	3.21773400	1.91586200
H	-0.59928500	3.26931100	0.24001700
H	-1.34827800	-0.90471300	-0.87299800
H	-1.67821500	-0.05583600	0.61722900
H	-4.23999200	1.53077200	-1.71480800
H	-3.74160200	-0.78011800	0.27028700
H	-6.34575500	0.45491100	-0.89734600
H	-5.75278500	-1.83312700	1.06463100
H	-8.18760200	-1.23666100	1.59801700
H	-8.39414000	-0.64360500	-0.06693800
H	-8.12445800	-2.37206200	0.25968400
H	1.35448000	-2.53341600	-0.18047300
H	4.56566700	-3.04854200	-0.54162300
H	4.51016300	-1.94840300	0.85259200
H	4.48829300	-0.74550300	-1.18712800
H	2.10519900	-1.94807100	-2.07217500
H	2.57908700	-2.81630900	1.41378100

Conformation 2 of compound **1** (Boltzmann distribution: 0.353)

Atom	x	y	z
O	1.10361000	0.85960500	1.92764600
C	1.65405600	-0.33172700	1.65709300
C	1.47453400	-0.62898600	0.14682900
C	2.67598100	0.03219200	-0.56190900
C	2.74760100	1.38887600	-0.61820400
C	1.64838300	2.19332900	-0.01107300
C	0.49891000	1.42477200	0.68467800

O	2.21113500	-1.01487300	2.49292800
C	3.84698400	2.20934500	-1.24111600
O	1.64610200	3.41661600	-0.05246300
C	-0.64096700	2.32419800	1.10225400
C	0.18039300	0.16430800	-0.15481200
C	-1.09906500	-0.57897800	0.23494800
C	-2.35821700	-0.05266500	-0.44940000
C	-3.63193700	-0.54492900	0.09623400
C	-4.83006700	-0.18195600	-0.42526400
C	-6.11288600	-0.62765100	0.06704000
H	0.12961500	0.42193800	-1.21514700
O	-2.28851600	0.70831600	-1.41898100
C	-7.28296900	-0.23902300	-0.48565500
C	-8.63885800	-0.66438200	-0.02247400
O	1.33496900	-2.00129300	-0.12635900
C	4.56315000	-1.69766800	-0.14430500
C	3.72925800	-0.89177200	-1.16267700
O	3.15755300	-1.71954000	-2.18461500
O	3.80822300	-2.58599200	0.69956200
H	3.41714200	2.95641100	-1.91435200
H	4.39205700	2.76019100	-0.46634000
H	4.56542300	1.61271300	-1.80077900
H	-1.38240200	1.76366400	1.67483700
H	-0.26558100	3.14453700	1.71661000
H	-1.12204200	2.74722200	0.21789900
H	-1.00025200	-1.62882100	-0.07020200
H	-1.25490300	-0.60766400	1.31938300
H	-3.58609000	-1.21887900	0.94858800
H	-4.83119600	0.49716500	-1.27741800
H	-6.11867000	-1.30506000	0.92073600
H	-7.25175000	0.43903400	-1.33936400
H	-9.18283800	-1.16839000	-0.83166000
H	-8.58451300	-1.34021700	0.83557300
H	-9.24291900	0.20851800	0.25655900
H	2.11987000	-2.46795400	0.25135600
H	5.25207200	-2.32109900	-0.72096800
H	5.14913300	-1.00425500	0.46787800
H	4.46679400	-0.28097900	-1.68423700
H	2.35392900	-2.13187300	-1.82475400
H	3.64680600	-2.16260800	1.56091800

Conformation 3 of compound **1** (Boltzmann distribution: 0.143)

Atom	x	y	z
O	-1.96158700	-1.35112700	1.67834000

C	-1.48169900	-0.10111400	1.70551400
C	-0.94083900	0.25671900	0.29686100
C	-2.15031100	0.79345500	-0.49630200
C	-3.13758000	-0.07011700	-0.85481200
C	-3.00119000	-1.51168400	-0.49529300
C	-1.75312300	-1.93288400	0.32022100
O	-1.51238500	0.60468200	2.69391200
C	-4.40122200	0.26478100	-1.60331500
O	-3.83952200	-2.33984400	-0.82576600
C	-1.62702700	-3.43005100	0.47755600
C	-0.54179500	-1.14374600	-0.22636100
C	0.83356400	-1.60784400	0.26243200
C	1.98508800	-1.00807000	-0.54677500
C	3.25669900	-0.81671800	0.16562000
C	4.36815400	-0.34876400	-0.45522700
C	5.64477300	-0.13666500	0.18646600
H	-0.54366600	-1.15638400	-1.31909900
O	1.84155000	-0.76333700	-1.74801100
C	6.72378300	0.34001700	-0.47258700
C	8.06804500	0.58228800	0.13422300
O	0.15598000	1.13479200	0.34640500
C	-2.32023600	3.23438000	0.36642900
C	-2.16024600	2.27971400	-0.83641600
O	-1.03577400	2.61739300	-1.65967100
O	-1.29505100	3.13210500	1.37082400
H	-4.50430300	-0.39054500	-2.47321300
H	-5.27436900	0.08282100	-0.96705500
H	-4.43977600	1.29845900	-1.94255100
H	-2.57181600	-3.85409400	0.82186200
H	-1.37929500	-3.88558600	-0.48520200
H	-0.84391500	-3.67375100	1.19789500
H	0.98211800	-1.41999200	1.32974400
H	0.91832200	-2.69346100	0.12394500
H	3.28265700	-1.06326100	1.22428400
H	4.29785700	-0.11087200	-1.51632000
H	5.72300100	-0.37509900	1.24700000
H	6.62086300	0.57213300	-1.53328500
H	8.35926800	1.63435000	0.01988300
H	8.08886700	0.32794700	1.19763000
H	8.83667500	-0.00696600	-0.38225500
H	-0.11965900	1.93660800	0.85328400
H	-2.27352500	4.25286900	-0.02872000
H	-3.30535800	3.08034700	0.81898300
H	-3.03013800	2.48757100	-1.46032400

H	-0.23586800	2.25684000	-1.24047500
H	-1.60265100	2.56056700	2.09630800

Conformation 4 of compound **1** (Boltzmann distribution: 0.030)

Atom	x	y	z
O	0.70156100	1.11372300	1.48179700
C	0.84908400	-0.20215300	1.27791700
C	1.15158500	-0.43793200	-0.22396500
C	2.68639500	-0.26760300	-0.34037300
C	3.22738600	0.97154400	-0.19085100
C	2.32077200	2.13550500	0.05131900
C	0.80789600	1.83529000	0.18768400
O	0.86580100	-1.01793700	2.18033900
C	4.68992900	1.32952800	-0.24613700
O	2.74820600	3.27811100	0.14826500
C	-0.04883600	3.07638200	0.26489200
C	0.46263100	0.77542600	-0.88760200
C	-0.99887200	0.60716500	-1.32680700
C	-2.05112500	0.15399200	-0.32801200
C	-3.43956900	0.19844500	-0.81365900
C	-4.48712900	-0.16668200	-0.03399300
C	-5.87158400	-0.13940900	-0.44577000
H	1.00867200	1.02952300	-1.80098100
O	-1.74851000	-0.23959000	0.80205800
C	-6.88436900	-0.50382600	0.37091400
C	-8.33350400	-0.49621600	0.00483100
O	0.67154200	-1.66099600	-0.72308700
C	3.53895700	-2.56592500	0.50971000
C	3.50791200	-1.51952700	-0.62528700
O	3.13829400	-2.08941100	-1.88907900
O	2.26032300	-3.09220300	0.90124000
H	4.84784400	2.14487400	-0.95826900
H	5.02492600	1.69615400	0.73059700
H	5.32775600	0.49439800	-0.53062500
H	-1.08782500	2.80987000	0.46853300
H	0.31094300	3.73711300	1.05588400
H	-0.00118100	3.62132800	-0.68164700
H	-1.33623700	1.54541400	-1.78306600
H	-1.01576200	-0.12207300	-2.14785100
H	-3.60648200	0.54517100	-1.83077700
H	-4.27452600	-0.50291400	0.98052400
H	-6.09038800	0.19297000	-1.46034400
H	-6.64114900	-0.83212500	1.38218000
H	-8.89966000	0.15488800	0.68325600

H	-8.49474600	-0.15525300	-1.02170500
H	-8.76409400	-1.50009700	0.11214500
H	1.02101500	-2.38316500	-0.14872400
H	4.12822500	-3.41155600	0.14448500
H	4.04946200	-2.13488500	1.37788700
H	4.55185700	-1.22962400	-0.74985300
H	2.16943500	-2.17018400	-1.90882900
H	1.90882600	-2.56743000	1.64604800

Conformation 5 of compound **1** (Boltzmann distribution: 0.012)

Atom	x	y	z
O	0.94061100	0.73195100	1.94977600
C	1.42807900	-0.47284700	1.62269000
C	1.35961100	-0.63407600	0.08300200
C	2.66257100	-0.01442600	-0.46625900
C	2.83741000	1.33185100	-0.39187700
C	1.74612100	2.16888300	0.18387700
C	0.48838800	1.43987600	0.71486300
O	1.86220600	-1.26151000	2.43813900
C	4.04443300	2.11272600	-0.84187300
O	1.83179100	3.38850200	0.24178300
C	-0.61815900	2.38833100	1.11257800
C	0.15463300	0.27738300	-0.25116500
C	-1.20027000	-0.39572400	-0.02090100
C	-2.36179800	0.26963800	-0.75498700
C	-3.70581900	-0.15677100	-0.33313000
C	-4.82436000	0.34185600	-0.91431000
C	-6.21435300	0.02467200	-0.61473200
H	0.20901400	0.61948700	-1.28727500
O	-2.16208000	1.08255600	-1.66193100
C	-6.65244400	-0.90818000	0.25747900
C	-8.09126100	-1.20331100	0.53717700
O	1.14621000	-1.96513800	-0.31711400
C	4.37778200	-1.92263800	-0.07308700
C	3.68992700	-0.96627600	-1.07039400
O	3.13953800	-1.65415400	-2.20182200
O	3.49346900	-2.80228200	0.64460200
H	3.74064400	2.90473300	-1.53290400
H	4.51230200	2.60690600	0.01659200
H	4.79866500	1.49846900	-1.33052500
H	-1.44171300	1.84338400	1.57799700
H	-0.24135100	3.13051500	1.81868800

H	-0.99299100	2.91043300	0.22973100
H	-1.14754000	-1.42492600	-0.39891000
H	-1.44402500	-0.49092200	1.04329300
H	-3.75961100	-0.88432700	0.47231500
H	-4.66966400	1.07802000	-1.70171000
H	-6.95467200	0.60301800	-1.16520300
H	-5.92800600	-1.50699500	0.80924800
H	-8.31515400	-1.05455900	1.60154900
H	-8.76077700	-0.57057200	-0.05224500
H	-8.31979000	-2.25468600	0.31983900
H	1.85980100	-2.52048500	0.08083400
H	5.04888200	-2.55933700	-0.65613500
H	4.97753300	-1.33718100	0.63141700
H	4.50944400	-0.37577900	-1.48146600
H	2.28313400	-2.03178200	-1.93823200
H	3.29895900	-2.42861900	1.52193800

ECD calculations data of **3**

Conformation 1 of compound **3** (Boltzmann distribution: 0.198)

Atom	x	y	z
C	-7.05953000	-2.88782300	2.25243000
C	-6.04519400	-1.84124700	1.88502300
C	-5.82291500	-1.37984100	0.64754400
C	-4.81295400	-0.32306800	0.28945500
C	-3.73718800	-0.86593900	-0.68918200
C	-2.77207200	0.19489600	-1.13399500
C	-1.39484600	0.18769800	-0.93525700
O	-3.36677000	1.20932400	-1.74823900
C	-0.62743600	1.32957400	-1.37511300
C	-0.63625200	-1.01178500	-0.38602600
C	0.68652700	-0.57868900	0.31329000
C	1.48237700	0.00175800	-0.90148600
C	0.99412800	-0.85395200	-2.09859800
C	-0.10547300	-1.82953100	-1.59322800
C	0.86092800	1.46957300	-0.95347900
O	-1.14476200	2.26013400	-2.04499000
C	0.60846500	0.55694700	1.36092800
C	3.01070000	-0.12774100	-0.74020700
C	3.65152300	-1.44431600	-1.12636000
O	3.69964300	0.78693300	-0.29767000
C	5.01571300	-1.71399700	-0.46703500
C	4.91748300	-2.07466800	0.99324600
C	5.55213800	-1.44509000	1.99011900
C	5.47562200	-1.82177100	3.44325800

O	1.40986300	-0.77180200	-3.23535900
C	-1.08915800	-2.26133100	-2.66788800
O	0.58429900	-2.97028100	-1.04856500
C	1.57090500	2.43777300	-1.90420300
C	0.88853100	1.91458200	0.58968600
O	-0.76421900	0.67276800	1.86489600
C	-0.19309800	2.84919200	1.18463100
C	-1.26974300	1.91838700	1.69707400
O	-2.41456800	2.18146600	2.00245600
C	1.50647400	0.31003400	2.56548100
O	2.11522100	2.52735100	0.91258200
C	-0.66539800	4.11061100	0.46272600
H	1.15640500	-1.46505300	0.74098300
H	-1.24207000	-1.62897600	0.27669100
H	-7.60496100	-3.24642100	1.37359800
H	-6.58021900	-3.74976700	2.73388000
H	-7.78941800	-2.49476400	2.97164300
H	-5.45547900	-1.43323100	2.70828800
H	-6.40747300	-1.78607100	-0.18107000
H	-5.31508300	0.52611600	-0.18919600
H	-4.32563700	0.05607200	1.19411000
H	-3.21511500	-1.70442500	-0.22874500
H	-4.23727500	-1.24875700	-1.58859200
H	-2.62798100	1.84634500	-2.02822300
H	2.94955900	-2.26274900	-0.93108400
H	3.76432000	-1.40915800	-2.21914800
H	5.47107500	-2.54936600	-1.01528500
H	5.67188700	-0.84860700	-0.60130300
H	4.28228800	-2.93141200	1.23077100
H	6.18366700	-0.58827500	1.74775400
H	6.46922400	-2.06558200	3.84058700
H	5.09390800	-0.98894300	4.04760000
H	4.82314100	-2.68650100	3.60148900
H	-1.83838200	-2.93521400	-2.24381000
H	-0.55442300	-2.79056200	-3.46312600
H	-1.59306600	-1.40887100	-3.12654800
H	0.81560800	-3.57243800	-1.77172600
H	1.50721600	2.06606700	-2.93069600
H	1.09569800	3.41883100	-1.87376100
H	2.62197400	2.55400600	-1.64546400
H	0.29191600	3.18777800	2.11355600
H	1.48602800	1.16136400	3.25011900
H	1.15856100	-0.58120300	3.09597200
H	2.54164500	0.14376200	2.25770300

H	2.84442700	2.01374900	0.50091400
H	-1.32537200	4.67610700	1.12637100
H	0.19500200	4.74289600	0.22741000
H	-1.20278000	3.89468300	-0.45954700

Conformation 2 of compound **3** (Boltzmann distribution: 0.093)

Atom	x	y	z
C	-7.84853400	-2.11457300	2.08676900
C	-6.93323800	-1.18996800	1.33418700
C	-5.59906500	-1.15970700	1.44639000
C	-4.69299100	-0.22166000	0.69452400
C	-3.70595100	-0.98961000	-0.22608100
C	-2.76749300	-0.07122600	-0.95438800
C	-1.38296300	-0.03722900	-0.82107400
O	-3.39654100	0.77275800	-1.76186300
C	-0.64323000	0.96473500	-1.55310200
C	-0.59560100	-1.07486700	-0.03417300
C	0.75738800	-0.49761500	0.47640700
C	1.49097900	-0.22935200	-0.87852400
C	0.94988500	-1.34582700	-1.80968300
C	-0.11878800	-2.16403800	-1.03121400
C	0.86319900	1.18864300	-1.24910800
O	-1.19657400	1.71447900	-2.39772300
C	0.72674900	0.85503600	1.22553900
C	3.02618200	-0.32962300	-0.76466700
C	3.64948800	-1.70704900	-0.84810000
O	3.73458000	0.65865900	-0.59610200
C	5.04391500	-1.82436000	-0.20682400
C	5.01584600	-1.81813000	1.30030100
C	5.69818600	-0.97328900	2.08359800
C	5.69156400	-0.98469300	3.58666500
O	1.30973800	-1.54278700	-2.95130200
C	-1.14837900	-2.84200200	-1.91987800
O	0.60297200	-3.14268100	-0.25937400
C	1.52264700	1.89337500	-2.43816900
C	0.96608000	1.98749200	0.14011000
O	-0.62167100	1.09978100	1.74993100
C	-0.08539800	3.04798500	0.54853800

C	-1.13423300	2.27761400	1.31951300
O	-2.26200200	2.61941700	1.61041200
C	1.68071000	0.89451300	2.41111600
O	2.20625000	2.64758900	0.24864100
C	-0.59590500	4.10632000	-0.42870700
H	1.25160200	-1.26025600	1.07902100
H	-1.16780900	-1.50713900	0.78607600
H	-7.28981600	-2.78781200	2.74485400
H	-8.56179100	-1.54978300	2.70064100
H	-8.44537200	-2.72553000	1.39765600
H	-7.41527500	-0.48972700	0.64898300
H	-5.10974300	-1.85772800	2.12937800
H	-5.28652700	0.47323300	0.09241700
H	-4.10780000	0.37988900	1.40192600
H	-3.15465300	-1.72515600	0.36019100
H	-4.28159600	-1.53973600	-0.98064000
H	-2.67564400	1.32329800	-2.21668700
H	2.95816700	-2.44660000	-0.42883200
H	3.71157400	-1.93867600	-1.92081400
H	5.47193600	-2.77325900	-0.55613500
H	5.69276100	-1.02527000	-0.57816900
H	4.39256900	-2.58378500	1.76840700
H	6.31875500	-0.20938600	1.61131600
H	5.34180800	-0.02466500	3.98732300
H	5.04546000	-1.77565000	3.98091200
H	6.70279200	-1.13872000	3.98448900
H	-1.86113900	-3.40177200	-1.30847000
H	-0.64503600	-3.54122700	-2.59553000
H	-1.69219800	-2.12625700	-2.53848500
H	0.80264100	-3.90297200	-0.82578900
H	1.41791300	1.28045600	-3.33781600
H	1.03994900	2.85239900	-2.62896100
H	2.58290200	2.06724300	-2.26344000
H	0.44681700	3.59228300	1.34443100
H	1.69020500	1.88490400	2.87239600
H	1.35982800	0.15911300	3.15472500
H	2.70052700	0.64991300	2.10464800
H	2.91600800	2.04690300	-0.06815700
H	-1.20891700	4.82944100	0.11624600
H	0.25169200	4.64391700	-0.86241000
H	-1.19289700	3.68501800	-1.23645000

Conformation 3 of compound **3** (Boltzmann distribution: 0.093)

Atom	x	y	z

C	7.70880000	-1.79517300	-2.06000500
C	6.56229800	-0.99149000	-1.51364900
C	6.11641400	-1.03665200	-0.25146400
C	4.97371600	-0.21919600	0.28732500
C	3.80951200	-1.11758400	0.78802800
C	2.68892900	-0.31731500	1.38597900
C	1.39141300	-0.22792900	0.89195800
O	3.05835500	0.36139200	2.46456700
C	0.45914800	0.65627800	1.55425900
C	0.86975700	-1.10043900	-0.24219100
C	-0.30640800	-0.41061200	-0.99313300
C	-1.38308400	-0.35507600	0.14283000
C	-1.07387500	-1.60922200	1.00011100
C	0.16292300	-2.32650700	0.39536500
C	-0.90981400	0.97165800	0.89182400
O	0.74508900	1.23605800	2.63276700
C	-0.09317000	1.04520300	-1.47035100
C	-2.83171400	-0.39788400	-0.39449100
C	-3.45283200	-1.75492600	-0.63894800
O	-3.44098200	0.62650700	-0.68740500
C	-4.81287600	-1.73551900	-1.35743900
C	-5.97795400	-1.38686400	-0.46588800
C	-6.88464900	-0.43315300	-0.71032000
C	-8.06745000	-0.11549700	0.16152800
O	-1.69632300	-1.96065900	1.98095200
C	0.93702800	-3.18076900	1.38431700
O	-0.33067700	-3.13190700	-0.69204400
C	-1.88645200	1.49399900	1.94817900
C	-0.63946200	1.99097400	-0.32114000
O	1.34357400	1.33302000	-1.55898000
C	0.46740000	3.06970600	-0.23919800
C	1.70011600	2.40436400	-0.80960300
O	2.85917800	2.75249600	-0.71744300
C	-0.68262500	1.30715200	-2.84963700
O	-1.81085300	2.69785500	-0.65456100
C	0.66745000	3.93666700	1.00308400
H	-0.60557000	-1.04722500	-1.82606700
H	1.65147700	-1.40442300	-0.93764400
H	7.38456700	-2.42528500	-2.89809400
H	8.49913400	-1.14088700	-2.44958300
H	8.14809800	-2.44308700	-1.29471600
H	6.06910900	-0.31804200	-2.21746300
H	6.60239500	-1.71029700	0.45797800
H	5.31744100	0.39560400	1.12796600

H	4.60022100	0.46404200	-0.48284500
H	3.45426400	-1.74199300	-0.03138500
H	4.18752000	-1.78838300	1.57015400
H	2.23093800	0.84522400	2.79549600
H	-2.71962500	-2.34918300	-1.20010900
H	-3.54104000	-2.26105300	0.33039800
H	-4.77605300	-1.05608100	-2.21531300
H	-4.96650200	-2.74629400	-1.75908200
H	-6.08358100	-1.99495200	0.43601100
H	-6.77294200	0.17494900	-1.61001800
H	-8.03844200	0.92718800	0.50311200
H	-9.00894200	-0.23668300	-0.38948500
H	-8.10314600	-0.76319100	1.04357500
H	1.77517600	-3.66985900	0.88054900
H	0.27949300	-3.95298000	1.79661900
H	1.31725100	-2.59497700	2.22272400
H	-0.68558300	-3.95937400	-0.33405300
H	-2.02407100	0.74481900	2.73282400
H	-1.49734300	2.39990300	2.41416400
H	-2.85910800	1.72051800	1.51384400
H	0.16975100	3.75381200	-1.04932000
H	-1.74544500	1.05553500	-2.87790300
H	-0.57276200	2.35780800	-3.12889300
H	-0.16253300	0.68765300	-3.58617900
H	-2.55929300	2.06153900	-0.69254700
H	1.39982000	4.71753600	0.78033200
H	-0.27520700	4.42554500	1.26354600
H	1.01641400	3.36931700	1.86461600

Conformation 4 of compound **3** (Boltzmann distribution: 0.086)

Atom	x	y	z
C	-6.67456400	3.98522300	-1.87373100
C	-5.84571900	2.78219500	-1.52035100
C	-5.56915800	2.37146800	-0.27592800
C	-4.74664300	1.15978700	0.06995700
C	-3.47590000	1.54102500	0.87711800
C	-2.69124900	0.33860200	1.31423700
C	-1.39608800	0.01678700	0.91998700
O	-3.37266800	-0.45307700	2.13179300
C	-0.81707400	-1.21866300	1.39528900
C	-0.51468400	0.95549000	0.10834600
C	0.55910800	0.16975800	-0.69979100
C	1.40862700	-0.44305800	0.46324100
C	1.29898300	0.61654900	1.58972200

C	0.36492500	1.75852400	1.10402600
C	0.52186800	-1.72663400	0.79375000
O	-1.39627400	-1.93931700	2.24803200
C	0.08133500	-1.02044800	-1.56480700
C	2.88509700	-0.66947900	0.07733400
C	3.84049100	0.49302800	0.22495500
O	3.28213400	-1.73853800	-0.37903600
C	5.16735900	0.31709400	-0.53037700
C	6.10889300	1.46666400	-0.28383000
C	6.57881600	2.29135300	-1.22854400
C	7.52704200	3.43356000	-0.99196400
O	1.85307500	0.55441600	2.66737000
C	-0.31405200	2.52333700	2.22749700
O	1.18112000	2.64287100	0.31277500
C	1.16898500	-2.72708300	1.75548100
C	0.20437700	-2.32104500	-0.66488500
O	-1.35064900	-0.88195400	-1.85225200
C	-1.12699100	-3.04936100	-0.97099900
C	-2.06344100	-1.96165700	-1.44817100
O	-3.27209400	-1.99520400	-1.55282200
C	0.80064300	-1.09944000	-2.90432600
O	1.20918700	-3.22001600	-1.07213600
C	-1.71416900	-4.09875600	-0.02787700
H	1.12433500	0.88125600	-1.30219900
H	-1.08484700	1.61681200	-0.54368800
H	-7.03173000	4.50541300	-0.97913600
H	-6.09783900	4.69750000	-2.47749900
H	-7.54694500	3.70051800	-2.47567000
H	-5.45018500	2.20741800	-2.36011100
H	-5.95933500	2.94331300	0.56905200
H	-5.34177900	0.46553300	0.67540100
H	-4.45342600	0.62704400	-0.84097600
H	-2.85977400	2.21980400	0.28799100
H	-3.77897600	2.07943900	1.78432200
H	-2.74616900	-1.21086800	2.38331400
H	3.32921200	1.41371100	-0.08104400
H	4.02818100	0.61065200	1.30134400
H	5.63189300	-0.62159200	-0.20481400
H	4.97018600	0.21724600	-1.60368600
H	6.42050400	1.61880500	0.75210400
H	6.26277900	2.13512600	-2.26195500
H	7.08885500	4.38491300	-1.31963800
H	7.79112200	3.52533600	0.06650500
H	8.45342900	3.30286900	-1.56578000

H	-0.95395400	3.30832300	1.81573600
H	0.44655000	2.99104200	2.86122200
H	-0.91292900	1.87169100	2.86596200
H	1.65411600	3.24630900	0.90512900
H	1.35630500	-2.24956000	2.72129600
H	0.50595900	-3.57633600	1.92415100
H	2.11535700	-3.09990000	1.36692300
H	-0.88028400	-3.57760300	-1.90529800
H	1.88360000	-1.14274500	-2.76585600
H	0.49308000	-1.98618900	-3.46365300
H	0.56432800	-0.20827600	-3.49311700
H	2.08572700	-2.83163700	-0.85766100
H	-2.57271800	-4.57358900	-0.51078800
H	-0.96759200	-4.87357600	0.16632000
H	-2.03860700	-3.68056300	0.92385200

Conformation 5 of compound **3** (Boltzmann distribution: 0.084)

Atom	x	y	z
C	6.79364500	-3.68104000	-1.65445600
C	5.85018400	-2.52674500	-1.46329300
C	5.67849300	-1.84784400	-0.32159800
C	4.74098400	-0.68439800	-0.14369200
C	3.65257300	-0.98078200	0.92390700
C	2.76744700	0.20374000	1.18136100
C	1.40420600	0.28392600	0.91408800
O	3.42610000	1.23905700	1.68587100
C	0.72339300	1.53293900	1.16630900
C	0.56918000	-0.91244700	0.47997600
C	-0.68514300	-0.46748400	-0.32992900
C	-1.47084400	0.32783900	0.76553900
C	-1.09177200	-0.39684300	2.08350500
C	-0.07550300	-1.52417800	1.75226900
C	-0.72915500	1.73596200	0.65707000
O	1.29405300	2.49858700	1.73526300
C	-0.46427800	0.51156200	-1.50740200
C	-2.99934400	0.30182300	0.55359300
C	-3.77575000	-0.85454800	1.14413300
O	-3.58087900	1.16829400	-0.09471000
C	-5.20209500	-1.03230500	0.59353000
C	-5.25564800	-1.53835600	-0.82547800
C	-5.77578500	-2.71659200	-1.19349300
C	-5.84117200	-3.23633300	-2.60239200
O	-1.52030000	-0.12114200	3.18465500
C	0.82519200	-1.90808400	2.91408600

O	-0.85282400	-2.65619600	1.31874100
C	-1.39425900	2.87160800	1.43923400
C	-0.65649000	1.97659600	-0.92977900
O	0.93406300	0.44862600	-1.94758600
C	0.52439500	2.73462800	-1.58352600
C	1.53720400	1.66173200	-1.91702600
O	2.71140200	1.79023800	-2.19600300
C	-1.32567400	0.18616100	-2.71960800
O	-1.81091400	2.63946400	-1.38993000
C	1.07319000	4.03954200	-1.00742600
H	-1.21575500	-1.35994000	-0.66282000
H	1.14546900	-1.65354400	-0.07302400
H	6.25521000	-4.58128000	-1.97713700
H	7.53149400	-3.46173300	-2.43674600
H	7.33371000	-3.91737000	-0.73201900
H	5.27100600	-2.22908900	-2.33964300
H	6.25215000	-2.14133600	0.56044900
H	5.30268000	0.20144500	0.17676600
H	4.25935400	-0.43588600	-1.09527800
H	3.06969100	-1.84942900	0.61870900
H	4.14326900	-1.23349700	1.87246400
H	2.73699800	1.96721000	1.84187100
H	-3.18930800	-1.77436300	1.03257600
H	-3.81809000	-0.66397800	2.22553300
H	-5.72553000	-1.73540700	1.25043900
H	-5.72612300	-0.07173100	0.66927200
H	-4.84059900	-0.88425700	-1.59353700
H	-6.19476500	-3.36631600	-0.42224400
H	-5.30863800	-4.19147300	-2.69526600
H	-6.87875300	-3.42682700	-2.90514700
H	-5.40191400	-2.52942600	-3.31355900
H	1.50529100	-2.70970400	2.61377200
H	0.21352600	-2.26534900	3.74892500
H	1.40926500	-1.06133500	3.27841400
H	-1.16040200	-3.14267000	2.09816300
H	-1.40181000	2.63451000	2.50675700
H	-0.84214300	3.80263000	1.30647100
H	-2.42169500	3.03012100	1.11513900
H	0.10708100	2.98372000	-2.57185700
H	-2.38468800	0.16095800	-2.45343900
H	-1.19014500	0.93243600	-3.50610300
H	-1.04417400	-0.79645500	-3.10914900
H	-2.59889000	2.22623300	-0.97284700
H	1.80658500	4.45623200	-1.70324900

H	0.26109800	4.76398900	-0.90228800
H	1.55088200	3.90700600	-0.03779700

Conformation 6 of compound **3** (Boltzmann distribution: 0.052)

Atom	x	y	z
C	7.11576100	-2.87666600	0.06279700
C	6.50199700	-1.85152500	-0.84945400
C	5.69451700	-0.85529000	-0.46268400
C	5.09629500	0.17202800	-1.38946900
C	3.55464200	0.14365500	-1.45617900
C	2.83688800	0.56756300	-0.20074300
C	1.50074700	0.28784400	0.07576600
O	3.55244900	1.32552000	0.61362100
C	0.89158800	0.90611500	1.22493600
C	0.66432900	-0.67442900	-0.75321300
C	-0.84222600	-0.27985900	-0.72605000
C	-1.17484000	-0.49872400	0.78686000
C	-0.24556100	-1.67051800	1.19576000
C	0.64678900	-2.04312800	-0.02152400
C	-0.65369300	0.88493600	1.38261800
O	1.56974700	1.53773800	2.07805000
C	-1.20486100	1.19079200	-1.03908500
C	-2.64922500	-0.87803300	1.03428000
C	-3.04186800	-2.33095200	0.86398800
O	-3.49497100	-0.04403400	1.34421600
C	-4.53664100	-2.56390000	0.57905100
C	-4.95000100	-2.18145700	-0.81906500
C	-5.92793600	-1.32067700	-1.12851500
C	-6.35888000	-0.96033300	-2.52271300
O	-0.22979500	-2.20611400	2.28418200
C	1.96993500	-2.68618900	0.35708000
O	-0.13534100	-2.93707800	-0.83653300
C	-1.01966100	1.13927700	2.84781900
C	-1.26120700	1.95565300	0.35001900
O	-0.11025900	1.82266000	-1.78562800
C	-0.51839100	3.27250900	0.01780200
C	0.35982900	2.93213600	-1.16598200
O	1.31403500	3.54276000	-1.60240900
C	-2.45862800	1.32036700	-1.89279300
O	-2.55332300	2.35678100	0.74338300
C	0.09990200	4.15125400	1.10415500
H	-1.38898000	-0.95959200	-1.38022800
H	1.02281500	-0.77204300	-1.77780000
H	6.81953200	-2.71761900	1.10464100

H	6.81781300	-3.89243000	-0.22708500
H	8.21160200	-2.84627500	0.01004700
H	6.75097700	-1.94125400	-1.90890700
H	5.44579700	-0.75983400	0.59545100
H	5.41638900	1.17709800	-1.09067400
H	5.46970400	0.00777500	-2.40601300
H	3.22017900	0.83149100	-2.24700600
H	3.21541900	-0.84980700	-1.75874600
H	2.94285700	1.55655000	1.39526700
H	-2.41587500	-2.79174700	0.09170800
H	-2.75934600	-2.82560200	1.80408100
H	-4.72284000	-3.63544500	0.73025500
H	-5.14368500	-2.02713000	1.31440900
H	-4.40318000	-2.67076200	-1.62862600
H	-6.47014800	-0.83286400	-0.31625000
H	-7.41696400	-1.20325600	-2.68379300
H	-6.25572500	0.11755400	-2.70184400
H	-5.76821200	-1.49043400	-3.27677600
H	2.53472600	-2.94297900	-0.54298200
H	1.78200600	-3.60431000	0.92328000
H	2.57757000	-2.03338800	0.98592100
H	-0.05736600	-3.83542900	-0.48171200
H	-0.56855100	0.37226700	3.48359400
H	-0.63859900	2.10846400	3.17153500
H	-2.09720700	1.12173300	3.00100000
H	-1.32091900	3.86909700	-0.44376600
H	-2.73035400	2.36940900	-2.03258100
H	-2.27390000	0.86826600	-2.87174800
H	-3.30528700	0.80549100	-1.43206300
H	-3.05487000	1.56696300	1.04216500
H	0.45298700	5.08290600	0.65343400
H	-0.66183200	4.40506700	1.84633800
H	0.93553100	3.67151000	1.61164700

Conformation 7 of compound **3** (Boltzmann distribution: 0.052)

Atom	x	y	z
C	6.37262000	0.50359400	-2.63299100
C	6.13257300	-0.55313500	-1.59073100
C	5.30222100	-0.43732400	-0.54553400
C	5.08987900	-1.51146000	0.48864900
C	3.62809500	-2.02256400	0.57354500
C	2.66521700	-1.09166500	1.25893100
C	1.41247600	-0.70935300	0.78459400
O	3.10329300	-0.66981100	2.43718800

C	0.59255200	0.14810800	1.61047900
C	0.81029900	-1.24975600	-0.50514100
C	-0.25700900	-0.27265400	-1.08395800
C	-1.34391200	-0.33174300	0.04186000
C	-1.20488900	-1.76638900	0.61042900
C	-0.05836200	-2.48573200	-0.14854600
C	-0.73138300	0.74127300	1.05123700
O	0.94158000	0.48078600	2.77249400
C	0.12906000	1.21848400	-1.24082500
C	-2.78026800	-0.09422600	-0.48148700
C	-3.52747600	-1.27823600	-1.05700400
O	-3.28337900	1.02495300	-0.50478800
C	-4.90569700	-0.95421200	-1.65859000
C	-5.99571900	-0.78086100	-0.63079900
C	-6.81950300	0.27012800	-0.53963500
C	-7.92613500	0.42066700	0.46682500
O	-1.87937000	-2.23654600	1.50315000
C	0.57176700	-3.63097600	0.62522200
O	-0.62264500	-2.96256900	-1.38472000
C	-1.65199200	1.12920800	2.21132800
C	-0.33971100	1.95811700	0.08089900
O	1.59008800	1.35784100	-1.26238600
C	0.86919500	2.87699700	0.38029500
C	2.03794000	2.21607400	-0.31398500
O	3.22409800	2.41812800	-0.15311400
C	-0.39559300	1.82821300	-2.53354800
O	-1.42471400	2.83878600	-0.09225700
C	1.13220000	3.45083200	1.77207100
H	-0.61103800	-0.67732200	-2.03186400
H	1.56348000	-1.49164400	-1.25495700
H	5.77353300	1.39989900	-2.44420500
H	7.42968700	0.79799500	-2.66076400
H	6.12671100	0.13159400	-3.63595800
H	6.69394100	-1.48211000	-1.71264800
H	4.73511800	0.48623500	-0.41901300
H	5.72685700	-2.37244200	0.25898800
H	5.38734800	-1.15077100	1.48061700
H	3.27161100	-2.29088500	-0.42110900
H	3.62765100	-2.94384200	1.17205300
H	2.34736800	-0.12704700	2.84460300
H	-2.86937300	-1.73625100	-1.80761500
H	-3.62648300	-2.03608200	-0.27119200
H	-4.84050200	-0.06719500	-2.29694800
H	-5.16650400	-1.79805800	-2.31154300

H	-6.11825500	-1.60763900	0.07326500
H	-6.69230800	1.09590400	-1.24216200
H	-7.98269400	-0.44470700	1.13508600
H	-7.78194100	1.31892700	1.08086100
H	-8.89899000	0.53457100	-0.02837800
H	1.36655100	-4.09263800	0.03375500
H	-0.18936500	-4.38886100	0.83791400
H	0.97911400	-3.30367300	1.58329400
H	-1.08922800	-3.79485100	-1.21692600
H	-1.87898800	0.24824100	2.81810700
H	-1.16696700	1.86483900	2.85358100
H	-2.58956000	1.54931100	1.85020500
H	0.65868700	3.73816000	-0.27323100
H	-0.16746800	2.89561600	-2.58306600
H	0.07072100	1.32475200	-3.38542600
H	-1.47856300	1.70720600	-2.61310500
H	-2.24135800	2.31067800	-0.23554700
H	1.94571300	4.17898200	1.71027600
H	0.24012200	3.97423400	2.12666300
H	1.40307700	2.68786800	2.50031300

Conformation 8 of compound **3** (Boltzmann distribution: 0.051)

Atom	x	y	z
C	-7.47396400	-2.87291300	1.81916500
C	-6.46888900	-2.32184300	0.84682900
C	-6.03860100	-1.05400400	0.81445500
C	-5.03960300	-0.50506600	-0.16830600
C	-3.77389500	0.03570100	0.54556900
C	-2.80182300	0.71439600	-0.37643300
C	-1.44048200	0.44533900	-0.47635500
O	-3.35683000	1.69161600	-1.08203900
C	-0.62600300	1.31237400	-1.29729300
C	-0.76566500	-0.74942500	0.17952200
C	0.72011000	-0.44069300	0.53167500
C	1.32768700	-0.25674700	-0.89794300
C	0.49181900	-1.22185700	-1.77627000
C	-0.61437900	-1.86136800	-0.89280200
C	0.92162900	1.25997000	-1.17233700
O	-1.13202800	2.17085900	-2.06384400
C	1.01925200	0.86931300	1.29788400
C	2.82336100	-0.62656600	-0.96886400
C	3.17915400	-2.08701100	-1.15269300
O	3.71000300	0.21602900	-0.86539900
C	4.59424400	-2.46941500	-0.68225300

C	4.73163700	-2.52860200	0.81760200
C	5.61113100	-1.82728300	1.54388500
C	5.76177500	-1.90553100	3.03741900
O	0.67901500	-1.43780300	-2.95543400
C	-1.84721000	-2.29891200	-1.66451200
O	-0.00332800	-2.98651700	-0.23339400
C	1.55559100	1.87972800	-2.42095100
C	1.32184900	1.98104300	0.20648000
O	-0.19116400	1.32507000	1.99168400
C	0.52420300	3.19003100	0.75294200
C	-0.54557500	2.58264700	1.63350000
O	-1.56058400	3.09747100	2.05586400
C	2.10025100	0.69911800	2.35619700
O	2.66145500	2.41651100	0.17297700
C	0.09700000	4.35453500	-0.13968900
H	1.13701600	-1.29991300	1.05809100
H	-1.30550600	-1.11536900	1.05193800
H	-7.81970500	-2.10558900	2.51916100
H	-8.34892900	-3.27821300	1.29514900
H	-7.04719700	-3.70027900	2.40031100
H	-6.07301200	-3.02691900	0.11324500
H	-6.42869600	-0.34427200	1.54743100
H	-4.75743400	-1.28221100	-0.88709600
H	-5.48998400	0.31585900	-0.73853900
H	-4.08251800	0.79396200	1.27880800
H	-3.29152200	-0.76642600	1.10400300
H	-2.62062000	2.08220400	-1.65929100
H	2.42007600	-2.71274300	-0.67003100
H	3.07815100	-2.28154900	-2.22989300
H	4.81020600	-3.45978100	-1.10406000
H	5.32698900	-1.77519800	-1.10478700
H	4.05508100	-3.21660600	1.33028700
H	6.28341200	-1.13921300	1.02788800
H	5.60078700	-0.92446000	3.50225400
H	5.05153400	-2.61302700	3.47737500
H	6.77561200	-2.22025800	3.31596800
H	-2.57609300	-2.74703700	-0.98363600
H	-1.56535800	-3.04625900	-2.41329600
H	-2.31501300	-1.46694500	-2.19353500
H	-0.00550600	-3.74393700	-0.83772000
H	1.23622100	1.33061100	-3.31117200
H	1.23838900	2.91680000	-2.53539900
H	2.64282700	1.85334400	-2.37330000
H	1.23143500	3.60620300	1.48748200

H	2.33897500	1.65417600	2.83042300
H	1.74552600	0.00192500	3.12092500
H	3.01684600	0.29344900	1.92168800
H	3.21524200	1.71180300	-0.22864500
H	-0.31855900	5.14972100	0.48538800
H	0.97047800	4.75814200	-0.65891200
H	-0.64908500	4.07035900	-0.88035700

ECD calculations data of **5**

Conformation 1 of compound **5** (Boltzmann distribution: 0.620)

Atom	x	y	z
C	1.38599200	0.56314500	0.50378600
C	1.75634800	1.84410100	-0.08827300
C	-0.02610600	0.55835400	1.05332900
C	-0.17715700	1.70760100	2.08043200
C	0.06980200	3.02625300	1.31703700
C	0.57325800	2.85456100	-0.11077100
C	-1.03759400	0.83377600	-0.11351600
C	-0.58901900	2.09771000	-0.90620800
C	0.78870400	1.58755500	3.26765900
O	-1.52888500	1.69729700	2.55486600
O	2.85083600	2.16143700	-0.56510400
C	2.19602400	-0.55254700	0.55378400
O	1.67327600	-1.63684100	1.18507000
C	3.52707800	-0.63611100	-0.01290400
C	4.39580900	-1.66494200	0.17287900
C	5.71185500	-1.71744400	-0.42426400
C	6.57650500	-2.73554100	-0.22251100
C	7.94527300	-2.83213600	-0.81690200
C	-1.18794800	-0.39743000	-1.00063100
C	-2.07797600	-1.48904200	-0.60784800
O	-0.51619300	-0.47689700	-2.05496000
O	-0.18244700	4.10473800	1.83195100
C	-1.76280900	2.98610400	-1.22573600
C	-2.16444500	3.29010800	-2.46637500
C	0.96522100	4.17498600	-0.76044800
C	-2.86993900	-1.45906200	0.56869900
C	-3.70661400	-2.49427600	0.95021200
C	-3.75805300	-3.62985500	0.10195200
C	-3.00401500	-3.70955800	-1.06627800
C	-2.16374100	-2.65276000	-1.42802800

C	-4.53821900	-2.44137800	2.20704900
O	-4.58477800	-4.63781400	0.49022500
O	-1.44942500	-2.77594900	-2.56407000
C	-3.35205000	4.14409600	-2.80728500
H	-0.25202800	-0.38595300	1.54851600
H	-2.00122000	1.03897100	0.35514000
H	-0.13086700	1.77504600	-1.84551000
H	0.61878100	2.41568200	3.96219500
H	0.60087500	0.64421100	3.78904400
H	1.83556900	1.61259600	2.95484300
H	-1.67930700	2.52156200	3.04347500
H	2.22995800	-2.41796200	1.05830800
H	3.82374000	0.21676400	-0.61073400
H	4.14343500	-2.51156800	0.81307400
H	6.00094000	-0.88483400	-1.06510600
H	6.26779000	-3.55964400	0.42196300
H	8.17757000	-1.97026900	-1.44895000
H	8.70583800	-2.90183500	-0.02849800
H	8.04304300	-3.74405200	-1.41991500
H	-2.32526600	3.37814300	-0.37591600
H	-1.59811700	2.89325300	-3.31073000
H	1.81850200	4.62032400	-0.24318400
H	1.24646100	4.01511700	-1.80458300
H	0.13354700	4.88058900	-0.72811400
H	-2.82481100	-0.58874100	1.21401900
H	-3.05592200	-4.58464900	-1.70725100
H	-4.28775900	-3.26316700	2.88736000
H	-4.37541900	-1.49892400	2.73583800
H	-5.60755300	-2.52865800	1.98383900
H	-4.55336400	-5.36933200	-0.14402400
H	-0.92223100	-1.93180200	-2.64547900
H	-3.86323200	4.50313800	-1.90857700
H	-3.04989200	5.01478600	-3.40287900
H	-4.07523500	3.58583700	-3.41504000

Conformation 2 of compound **5** (Boltzmann distribution: 0.239)

Atom	x	y	z
C	1.37313900	0.61001400	0.55293700
C	1.73009500	1.87808300	-0.07466900
C	-0.05173200	0.59336700	1.06951500
C	-0.24273400	1.76232300	2.06769600
C	0.00679200	3.06774200	1.28324400
C	0.53493000	2.87338600	-0.13169500
C	-1.04224100	0.82491700	-0.12453300

C	-0.60295900	2.08361300	-0.93102500
C	0.69263800	1.67842600	3.28183700
O	-1.60646200	1.74519700	2.50721900
O	2.82036200	2.19524100	-0.56108700
C	2.20433000	-0.48628600	0.66279800
O	1.66146800	-1.58294800	1.25386500
C	3.57363400	-0.53740800	0.19134500
C	4.35572500	-1.64879400	0.16343500
C	5.72181500	-1.65222200	-0.31070700
C	6.48845500	-2.76370300	-0.35328400
C	7.90254300	-2.81542900	-0.83671100
C	-1.14282600	-0.42601100	-0.99080800
C	-2.02047700	-1.52865300	-0.60115900
O	-0.43958800	-0.51190800	-2.02389900
O	-0.26241000	4.15386900	1.77327300
C	-1.78548900	2.94767400	-1.28313600
C	-2.17230600	3.22507700	-2.53467700
C	0.91930700	4.18531700	-0.80280500
C	-2.84716000	-1.49235100	0.55105100
C	-3.67257500	-2.53760500	0.92964000
C	-3.67528900	-3.69083700	0.10398900
C	-2.88543600	-3.77771700	-1.03983600
C	-2.05713000	-2.71054700	-1.39877100
C	-4.54147800	-2.47728100	2.16069600
O	-4.49200800	-4.70824200	0.48884900
O	-1.30657500	-2.84129800	-2.51028800
C	-3.36693300	4.05551300	-2.90774200
H	-0.27379800	-0.34282100	1.58158000
H	-2.02107100	1.01692500	0.31702300
H	-0.12470200	1.75348500	-1.85759700
H	0.49651200	2.52021900	3.95259300
H	0.50010700	0.74522900	3.81952200
H	1.74691100	1.70708400	2.99569200
H	-1.77687000	2.57386900	2.98150500
H	2.32141400	-2.27906100	1.37848700
H	3.96423700	0.40386800	-0.17496300
H	3.97823000	-2.61921800	0.48955200
H	6.13365600	-0.70109900	-0.64720000
H	6.05740300	-3.70598900	-0.01252900
H	8.26030600	-1.83315600	-1.15813700
H	8.56853400	-3.18917500	-0.04832400
H	7.99904400	-3.51446200	-1.67751200
H	-2.36701600	3.34519800	-0.44883700
H	-1.58710400	2.82293000	-3.36352700

H	1.75817900	4.65305200	-0.28171200
H	1.21935300	4.00776400	-1.83873900
H	0.07780600	4.87990100	-0.79796300
H	-2.83995300	-0.60859900	1.17942600
H	-2.89967600	-4.66651200	-1.66364400
H	-4.41322800	-1.52133600	2.67453000
H	-5.60187500	-2.59120100	1.90873300
H	-4.29431400	-3.28002800	2.86451900
H	-4.42690400	-5.45153300	-0.12894800
H	-0.79546600	-1.98743800	-2.59377200
H	-3.89660800	4.42217300	-2.02291400
H	-3.06842400	4.92039100	-3.51356100
H	-4.07279800	3.47657400	-3.51649900

Conformation 3 of compound **5** (Boltzmann distribution: 0.037)

Atom	x	y	z
C	1.38512200	0.56746900	0.51772100
C	1.75675200	1.84958200	-0.07091700
C	-0.03102100	0.55907900	1.05671100
C	-0.19469400	1.70962700	2.08012200
C	0.05171600	3.02767500	1.31563600
C	0.56978600	2.85512200	-0.10685400
C	-1.03406100	0.82913200	-0.11908300
C	-0.58136400	2.09138000	-0.91205800
C	0.76330500	1.59663400	3.27447800
O	-1.54962400	1.69379000	2.54503600
O	2.85496800	2.17108100	-0.53622400
C	2.19591600	-0.54764800	0.56855600
O	1.67108600	-1.63414800	1.19418400
C	3.52858600	-0.62884600	0.00545600
C	4.39619400	-1.65945100	0.18654700
C	5.71259900	-1.70948800	-0.41004100
C	6.57593700	-2.73006600	-0.21534900
C	7.94444000	-2.82425700	-0.81076700
C	-1.17234500	-0.40641600	-1.00171800
C	-2.06775300	-1.49693200	-0.61534900
O	-0.48377900	-0.49307600	-2.04393600
O	-0.21155600	4.10598400	1.82535500
C	-1.75452800	2.97478400	-1.24712400
C	-2.14462000	3.27258700	-2.49292700
C	0.96255300	4.17585600	-0.75544100
C	-2.88322400	-1.45382000	0.54205200
C	-3.72633000	-2.48715600	0.91990900
C	-3.75429100	-3.63560900	0.08690600

C	-2.97429200	-3.72741200	-1.06290100
C	-2.13291900	-2.67330500	-1.42198900
C	-4.58602200	-2.40459900	2.15898800
O	-4.54648500	-4.70555300	0.36849700
O	-1.39406500	-2.80706900	-2.54155100
C	-3.33103500	4.12206700	-2.84888700
H	-0.25810800	-0.38508100	1.55175800
H	-2.00158200	1.03450200	0.34102800
H	-0.11202600	1.76667100	-1.84514000
H	0.58396500	2.42505500	3.96627600
H	0.57657600	0.65321200	3.79613500
H	1.81227400	1.62648500	2.96926900
H	-1.70784400	2.51914800	3.02933600
H	2.22891700	-2.41453200	1.06789100
H	3.82704600	0.22699100	-0.58723000
H	4.14232800	-2.50979000	0.82121800
H	6.00283300	-0.87293600	-1.04521400
H	6.26621500	-3.55831100	0.42329900
H	8.17783200	-1.95805200	-1.43645200
H	8.70514800	-2.90100800	-0.02316600
H	8.04063600	-3.73176500	-1.42065400
H	-2.32647000	3.36889600	-0.40461600
H	-1.56890500	2.87382100	-3.32999400
H	1.80862200	4.62590700	-0.23048000
H	1.25512600	4.01501900	-1.79630600
H	0.12758900	4.87794700	-0.73310200
H	-2.85511100	-0.57504600	1.17667200
H	-3.01817100	-4.61726600	-1.68114300
H	-4.41546300	-1.45920300	2.67903800
H	-5.65673800	-2.45573300	1.92256500
H	-4.36342000	-3.20943700	2.87135000
H	-5.04729100	-4.56202400	1.18449000
H	-0.87081100	-1.96152100	-2.62477100
H	-3.85167300	4.48362400	-1.95663400
H	-3.02533000	4.99100000	-3.44522700
H	-4.04696500	3.55942100	-3.46115700

Reference

1. Abe, N.; Murata, T.; Hirota, A., Novel oxidized sorbicillin dimers with 1,1-diphenyl-2-picrylhydrazyl-radical scavenging activity from a fungus. *Biosci. Biotechnol. Biochem.* **1998,** 62, 2120-2126.
2. Kahlert, L.; Bassiony, E. F.; Cox, R. J.; Skellam, E. J., Diels–Alder Reactions During the Biosynthesis of Sorbicillinoids. *Angew. Chem. Int. Edit.* **2020,** 59, 5816-5822.
3. Ren, S.; Zeng, Y.; Wang, Q.; Lin, Q.; Yin, X.; Chen, S.; Wang, M.; Liu, L.; Gao, Z., Major Facilitator Superfamily Transporter Participates in the Formation of Dimeric Sorbicillinoids Pigments. *J.Agric.Food Chem.* **2023,** 71,

12216-12224.
